# Correction: Impact of genetic background as a risk factor for atherosclerotic cardiovascular disease: A protocol for a nationwide genetic case-control (CV-GENES) study in Brazil

**DOI:** 10.1371/journal.pone.0321003

**Published:** 2025-03-25

**Authors:** Haliton Alves de Oliveira Junior, Precil Diego Miranda de Menezes Neves, Gustavo Bernardes de Figueiredo Oliveira, Frederico Rafael Moreira, Maria Carolina Tostes Pintão, Viviane Zorzanelli Rocha, Cristiane de Souza Rocha, Viviane Nakano, Elisa Napolitano Ferreira, Diana Rojas-Málaga, Celso Ferraz Viana, Fabiula Fagundes da Silva, Juliete Jorge Vidotti, Natalia Mariana Felicio, Leticia de Araújo Vitor, Karina Gimenez Cesar, Camila Araújo da Silva, Lucas Bassolli de Oliveira Alves, Álvaro Avezum

The eighth author’s name is incorrect. The correct name is: Viviane Nakano.
