# Supplementary material for: Impact of genetic background as a risk factor for atherosclerotic cardiovascular disease: A protocol for a nationwide genetic case-control (CV-GENES) study in Brazil
Source: PLoS One. 2024 Mar 13;19(3):e0289439. doi: 10.1371/journal.pone.0289439 (PMC10936812; doi:10.1371/journal.pone.0289439)
Supplement: S2 File — (PDF) [file pone.0289439.s002.pdf]

CV-Genes

Avaliação do Impacto do Componente Genético como Fator de Risco para Doença Cardiovascular Aterosclerótica na População Brasileira

Versão 2.0 de 8 de novembro de 2022

Este documento é confidencial e seu uso, reprodução, divulgação e publicação são restritos aos principais pesquisador es e ao patrocinador

Declaração de Conformidade

Este documento é um protocolo específico elaborado para condução de um projeto de pesquisa, aqui também denominado "estudo". Como tal, será conduzido em conformidade com todas as premissas e conteúdo deste protocolo, assim como às normativas vigentes do Sistema CEP/CONEP e as diretrizes estabelecidas pelo Documento das Américas e pelo Guia de Boas Práticas Clínicas (E6/R2) do ICH – Conselho Internacional sobre Harmonização de Requisitos Técnicos em Produtos Farmacêuticos para Uso Humano.

• Rua Treze de Maio, 1.815 • Paulista
• CEP 01327 001 • São Paulo • SP
• Fone: 11 3549 1976 • Fax: 11 3549 0394

• Rua Treze de Maio, 1.815 • Paulista
• CEP 01327 001 • São Paulo • SP
• Fone: 11 3549 1976 • Fax: 11 3549
0394

SUMÁRIO

1. INFORMAÇÕES DO ESTUDO 5

1.1 Sinopse do protocolo 5

1.2 Histórico do protocolo 7

1.3 Contribuições 8

2. INTRODUÇÃO 8

3. OBJETIVOS 11

3.1. Objetivo Geral 11

3.2. Objetivo Específico 11

3.3. Objetivo(s) do Plano Nacional de Saúde ao(s) qual(is) o projeto se vinculará 12

4. POLÍTICA(S) PÚBLICA(S) VINCULADA(S) 12

5. POSSÍVEIS RISCOS NA EXECUÇÃO DO PROJETO 12

5.1. O projeto possui desafio (s) pertinente (s) ao desenvolvimento do sus: 16

5.2. Quais os benefícios ao sus: 16

6. JUSTIFICATIVA E RELEVÂNCIA DO PROJETO PARA O SUS 17

6.1. Fatores de Risco 17

6.2. Fatores Genéticos e Doença Cardiovascular 19

6.3. Contribuições esperadas 24

6.4. Custo-Efetividade da metodologia a ser empregada 25

7. EXPERTISE DO HOSPITAL DE EXCELÊNCIA PARA EXECUTAR O PROJETO 25

8. METODOLOGIA 27

8.1. Delineamento do estudo 27

8.2. Contexto 27

8.3. Participantes 29

8.4. Variáveis e Desfechos 30

8.5. Fonte de dados e mensuração 30

• Rua Treze de Maio, 1.815 • Paulista
• CEP 01327 001 • São Paulo • SP
• Fone: 11 3549 1976 • Fax: 11 3549
0394

8.5.1. Extração de dna das amostras 31

8.5.2. Construção das bibliotecas e sequenciamento do exoma e genoma low pass 1x 31

8.5.3. Análise dos dados gerados 32

8.5.4. Calibração e cálculo de PRS para doenças cardiovasculares 33

8.5.5. Seleção e avaliação de modelos PRS 34

8.5.6. Seleção dos SNPs para cálculo de PRS 35

8.5.7. Cálculo e calibração de PRS 35

8.5.8. Controle de viés 43

8.5.9. Tamanho amostral 43

8.5.10. Variáveis quantitativas e análise estatística 44

9. QUESTÕES ÉTICAS 45

10. DIVULGAÇÃO DOS RESULTADOS E MONITORAMENTO 46

11. CRONOGRAMA DE ENTREGAS, ATIVIDADES E MARCOS 47

12. ESTIMATIVA DE CUSTO POR ENTREGA 52

13. REFERÊNCIAS 54

• Rua Treze de Maio, 1.815 • Paulista
• CEP 01327 001 • São Paulo • SP
• Fone: 11 3549 1976 • Fax: 11 3549 0394

1. INFORMAÇÕES DO ESTUDO

1.1 Sinopse do protocolo

Categoria dos Dados	Informação
Patrocinador Principal	Ministério da Saúde – PROADI-SUS
Contato para consultas públicas/científicas	11-35490729/31997199394<br>aavezum@haoc.com.br<br>haoliveira@haoc.com.br<br>gfoliveira@haoc.com.br
Título público/científico	Avaliação do impacto do componente genético como fator de risco para doença cardiovascular aterosclerótica na população brasileira
Condição(s) ou problema (s) de saúde estudado (s)	Evento cardiovascular aterosclerótico (Infarto Agudo do Miocárdio, Acidente Vascular Encefálico e Eventos Trombótico-Isquêmicos Arteriais Periféricos)
Critérios de Inclusão	Indivíduos maiores de 18 anos. Os casos (N = 1867) serão selecionados pela ocorrência do primeiro evento cardiovascular aterosclerótico (Infarto Agudo do Miocárdio, Acidente Vascular Encefálico e Eventos Trombótico-Isquêmicos Arteriais Periféricos) em cerca de 50 Centros que prestam atendimento em saúde, incluindo para doenças cardiovasculares. Serão considerados os casos com 24 horas do início dos sintomas (ideal), e até cinco dias do início dos sintomas (aceitável). A proporção entre casos e controles será de 1:1. Os controles (N = 1867) serão indivíduos adultos maiores de 18 anos que procuraram atendimento médico nos mesmos locais por outras questões clínicas (sem DCV) ou indivíduos sem qualquer doença aterosclerótica manifesta.
Critérios de Exclusão	Será considerado inelegível para o estudo o participante que, porventura, estiver participando de qualquer outro

• Rua Treze de Maio, 1.815 • Paulista
• CEP 01327 001 • São Paulo • SP
• Fone: 11 3549 1976 • Fax: 11 3549 0394

	projeto de pesquisa vinculado ao Programa Genomas Brasil, seja atrelado ao PROADI-SUS ou não. Essa informação será largamente difundida no momento de treinamento dos centros de pesquisa. Essa atitude visa evitar duplicidades no banco de dados do projeto genomas Brasil.
Tipo de Estudo	Caso-controle não pareado
Tamanho de amostra	3734 participantes
Objetivo Geral	Avaliar o risco atribuível populacional associado à presença de polimorfismos em genes relacionados a doenças cardiovasculares e seu impacto como fator de risco independente para ocorrência de IAM, AVE e eventos trombótico-isquêmicos em território arterial periférico na população brasileira.
Objetivos Específicos	● Realizar genotipagem de pacientes com e sem doença cardiovascular manifesta, analisando polimorfismos associados às doenças cardiovasculares;<br>● Avaliar o impacto de polimorfismos genéticos como fator de risco não modificável para doença cardiovascular aterosclerótica;<br>● Avaliar a correlação entre os achados genéticos e fatores fenotípicos e comportamentais [Fenotípicos: (hipertensão arterial, dislipidemia, obesidade abdominal, diabetes, envelhecimento populacional e fatores bioquímicos como relação apolipoproteína B/A1, entre outros). Sócio-comportamentais: baixa escolaridade, tabagismo, falta de atividade física regular (sedentarismo), alterações psicossociais (estresse e depressão),dieta inadequada, consumo de bebida alcoólica] na ocorrência de DCV aterosclerótica;<br>● Analisar o perfil genômico

• Rua Treze de Maio, 1.815 • Paulista
• CEP 01327 001 • São Paulo • SP
• Fone: 11 3549 1976 • Fax: 11 3549 0394

	associado à ocorrência de DCV aterosclerótica da população brasileira em comparação àquele de outras populações conforme descrito em estudos publicados.
Aspectos Éticos	O presente estudo irá seguir todas as resoluções e normativas de ética em pesquisa com seres humanos, conforme estabelecido na Resolução CNS 466 de 2012. Todos os participantes deverão assinar o Termo de Consentimento Livre e Esclarecido (TCLE), de forma voluntária, e conduziremos o estudo respeitando todos os direitos dos participantes de pesquisa, incluindo o sigilo e a confidencialidade. A todos os participantes serão explicados os objetivos, riscos e benefícios esperados, além de todos os direitos com a participação no estudo. Será assegurado o direito de retirada de consentimento a qualquer momento.<br>Em um cenário em que o risco cardiovascular na sua natureza poligênica, a DCV pode ter pouca associação com o background genético, divulgar os dados previamente, sem os devidos ajustes por variáveis fenotípicas, clínicas e demográficas pode ser precipitado, podendo gerar alarde, desistências e até falta de credibilidade na presente pesquisa. Sendo assim, os dados de risco atribuível à genética só serão divulgados ao final do estudo, quando estiverem estatisticamente ajustados pelos fatores de risco cardiovascular tradicionais (ver seção fonte de dados e mensuração acima).<br>O projeto será submetido ao CEP do HAOC como centro coordenador ético.

1.2 Histórico do protocolo

**Protocolo**

• Rua Treze de Maio, 1.815 • Paulista
• CEP 01327 001 • São Paulo • SP
• Fone: 11 3549 1976 • Fax: 11 3549 0394

Versão	Data Final
2.0	08 de novembro de 2022

1.3 Contribuições

Nome do Autor	Função
Álvaro Avezum Júnior	Pesquisador Principal
Haliton Alves de Oliveira Júnior	Gerente de pesquisa
Gustavo Bernardes de Figueiredo Oliveira	Pesquisador
Precil Diego Miranda de Menezes Neves	Pesquisador
Fabíula Fagundes da Silva	Pesquisadora
Juliete Jorge Vidotti	Pesquisadora
Camila Araújo Silva	Coordenadora de Projetos
Antônio José Cordeiro Mattos	Coordenador de pesquisa
Karina Gimenez Cezar	Analista de projetos
Letícia de Araújo Vitor	Assistente administrativo

2. INTRODUÇÃO

As doenças cardiovasculares (DCV) são responsáveis por mais de 300.000 mortes ao ano, representando a principal causa de morte no Brasil, seguidas de neoplasias, doenças respiratórias e diabetes. Juntas, as doenças crônicas não transmissíveis (DCNT) são responsáveis por aproximadamente 70% das causas de morte em ambos os sexos.

Atualmente sabe-se que sete em cada dez casos de DCV podem ser explicados por fatores de risco como hipertensão arterial, baixa escolaridade, tabagismo, dislipidemia, dieta inadequada, obesidade abdominal, falta de atividade física regular, diabetes, alterações psicossociais, e poluição do ar - todos os quais podem ser modificados. Entretanto, nos últimos anos, o avanço nas tecnologias de sequenciamento genético associado à maior acessibilidade aos mesmos pela redução de custos, propiciou a identificação de polimorfismos genéticos associados ao maior risco de DCV, reforçando o

• Rua Treze de Maio, 1.815 • Paulista
• CEP 01327 001 • São Paulo • SP
• Fone: 11 3549 1976 • Fax: 11 3549 0394

modelo de interação genética e meio ambiente como deflagrador das DCV, sendo diferente dos outros fatores de risco conhecidos, fator não modificável.

Alguns estudos mostraram que, quando avaliados conjuntamente aos outros fatores de risco cardiovascular (APO B/A, hipertensão, diabetes, obesidade abdominal, tabagismo, sedentarismo, alcoolismo e depressão), treze polimorfismos foram associados a maior risco cardiovascular, sendo 11 desses relativos aos níveis séricos de APO B/A, 1 associado ao receptor de LDL e 1 à Apolipoproteína E. Entretanto, como já mencionado, não há dados exclusivamente de indivíduos brasileiros com sistematização da obtenção de informações sobre fatores de risco tradicionais e, desse modo, recomendáveis para uma análise integrada com objetivo de avaliar a contribuição do escore de risco genético sobre o impacto no risco atribuível populacional para a ocorrência de eventos ateroscleróticos em seu amplo espectro clínico.

Ademais, em função de sua diversidade étnica, miscigenação, múltiplas ancestralidades e alta prevalência de casamentos consanguíneos, a população brasileira possui peculiaridade genética e que pode refletir na predisposição a doenças, dentre elas, as cardiovasculares.

Este projeto tem como objetivo principal a avaliação da informação genômica previamente associada às DCV e sua importância como preditor de risco independente (expresso em *Odds Ratio*) e em conjunto com fatores de risco tradicionais (tabagismo, diabetes, hipertensão arterial, obesidade, ansiedade e depressão, dieta inadequada, sedentarismo, consumo moderado/elevado de bebida alcoólica e relação apolipoproteína B/A1 (ApoB/ApoA1) (1,2).

Será realizado um estudo caso-controle não pareado de indivíduos maiores de 18 anos. Os casos (N = 1867) serão selecionados pela ocorrência do primeiro evento cardiovascular aterosclerótico (Infarto Agudo do Miocárdio, Acidente Vascular Encefálico e Eventos Trombótico-Isquêmicos Arteriais Periféricos) em até 50 centros participantes que prestam assistência em saúde, incluindo para DCV. A proporção entre casos e controles será de 1:1. Os controles (N = 1867) serão indivíduos adultos maiores de 18 anos que procuraram atendimento médico nos mesmos locais por outras questões clínicas (sem DCV) ou indivíduos sem qualquer doença manifesta. A avaliação genética será realizada por meio da associação de *Low-covering Whole Genome Sequencing* (cobertura 1x) e *Whole Exome Sequencing* (média de cobertura 30x).

A análise de variantes e o resultado de testes genéticos serão realizados em parceria entre laboratório Fleury S.A e HAOC, salientando que o pesquisador principal de cada centro participante e/ou pesquisador assistente será responsável por informar os resultados aos pacientes sempre que solicitado. Cabe ressaltar que a base de dados gerada será compartilhada com o Ministério da Saúde para compor

a base de dados genômicos do Programa Nacional de Genômica e Saúde de Precisão - Genomas Brasil. Além disso, todas as amostras advindas de teste genético bem como o grande volume de dados gerados a partir delas serão armazenados durante todo o período do estudo e fornecidas ao Ministério da Saúde, sempre que solicitadas. Ressalta-se que o presente projeto e as parcerias nele estabelecidas estarão de acordo com as políticas e diretrizes do Programa Nacional de Genômica e Saúde de Precisão - Genomas Brasil.

Como o escopo do projeto está focado na construção de um *poligenic risk score* para variantes associadas à doença cardiovascular, tais informações de cunho genético serão divulgadas aos pacientes apenas após a finalização e publicação do estudo, quando o risco atribuível exclusivamente a dados genéticos na população brasileira será conhecido e ajustado por variáveis demográficas e fenotípicas. Conforme Manual de Pendências da CONEP, página 39: "O resultado de qualquer exame, não somente os de natureza genética, deve ser assegurado ao participante de pesquisa sempre que solicitado por ele, salvo quando essa informação interferir no desfecho da pesquisa". Nesse sentido, por tratar-se de condição de saúde de influência poligênica, o compartilhamento apenas dos testes genéticos é pouco conclusivo, sem o ajuste para variáveis clínicas, e pode gerar alarde, recusas e desistências quanto à participação na pesquisa. Da mesma forma, em relação a achados secundários ou incidentalomas nos testes genéticos, o Parecer Técnico da Sociedade Brasileira de Genética Médica e Genômica sobre testes genéticos (3) recomenda que "*informações sobre: achados secundários, variantes em heterozigose para doença recessiva (status de portador), farmacogenômica, entre outros*" devam constar no laudo do exame apenas se o paciente tenha requerido e consentido em Termo de Consentimento Livre e Esclarecido ser informado sobre tais condições. Mesmo assim, sempre que solicitado, os testes serão compartilhados, conforme legislação ética aplicável, porém não será prática do projeto a divulgação espontânea de achados genéticos sem o devido ajuste por variáveis clínicas. A comunicação dos resultados será feita diretamente pela equipe do projeto aos participantes envolvidos e, caso seja necessário devido aos resultados encontrados, o aconselhamento genético será aplicado.

Diante de um modelo de doença complexa ou poligênica a ser estudado, i.e., a presença de variantes em heterozigose somadas entre si para representar potencial patogênico, os cálculos estatísticos de tamanho amostral assumem um padrão de herança dominante, com desenho de estudo caso-controle não-pareado 1:1, uma amostra de 1867 casos e de 1867 controles teria poder estatístico de 90% para detectar OR genético de 1.3, assumindo frequência alélica em 10% neste caso-controle não-pareado 1:1 de fator genético. Considerou-se prevalência de 10% da doença (IAM/AVC/DAP) na população alvo, nível de significância alfa de 5%, teste de hipótese bilateral. Cálculo de tamanho amostral foi efetuado via

• Rua Treze de Maio, 1.815 • Paulista
• CEP 01327 001 • São Paulo • SP
• Fone: 11 3549 1976 • Fax: 11 3549 0394

QUANTO versão 1.2.4 (4,5). Considerando que, de acordo com os dados do IBGE, a região sudeste concentra 42% da população brasileira, nordeste 27%, sul 14%, norte 9%, e centro-oeste 8%, e que o número de casos previsto é de 1867, o N amostral por região será ajustado, preferencialmente, para 784 participantes de pesquisa na região Sudeste, 504 n Nordeste, 262 no Sul, 168 no Norte, e 149 na região Centro-Oeste. Para os controles será respeitada a mesma proporção.

O teste de equilíbrio genético de Hardy-Weinberg será avaliado no grupo controle via teste Qui-quadrado ou teste exato de Fisher. Para estimar a associação entre os polimorfismos genéticos e risco de doença cardiovascular, análises de regressão logística não condicional univariada e múltipla serão conduzidas. Regressão múltipla será aplicada via eliminação *backward* (a partir dos resultados da regressão univariada) ou construindo *full model* a partir de covariáveis de interesse específico. *Odds ratios* brutos e ajustados serão reportados com IC95%. Risco atribuível populacional será estimado.

Na caracterização da população do estudo, variáveis categóricas serão descritas com números e proporções. As variáveis quantitativas de distribuição normal e assimétrica serão descritas como média (desvio padrão) ou mediana (intervalo interquartil), respectivamente. A normalidade será avaliada por inspeção visual de histogramas e testes de normalidade. Comparação destas variáveis quantitativas contínuas serão efetuadas com teste t-pareado ou alternativa não paramétrica, teste dos postos assinalados de Wilcoxon.

Análise estatística será efetuada com o pacote estatístico SAS 9.4 (SAS Institute Inc, Cary, NC). Todos os testes de hipótese serão bilaterais, e valor-p < 0.05 será considerado significativo.

3. OBJETIVOS

3.1. Objetivo Geral

Avaliar o risco atribuível populacional associado à presença de polimorfismos em genes relacionados a DCV e seu impacto como fator de risco independente para ocorrência de IAM, AVE e eventos trombótico-isquêmicos em território arterial periférico na população brasileira.

3.2. Objetivo Específico

- Realizar genotipagem de pacientes com e sem doença cardiovascular manifesta, analisando polimorfismos associados às doenças cardiovasculares;

• Rua Treze de Maio, 1.815 • Paulista
• CEP 01327 001 • São Paulo • SP
• Fone: 11 3549 1976 • Fax: 11 3549 0394

- Avaliar o impacto de polimorfismos genéticos como fator de risco não modificável para doença cardiovascular aterosclerótica;
- Avaliar a correlação entre os achados genéticos e fatores fenotípicos e comportamentais (Fenotípicos: (hipertensão arterial, dislipidemia, obesidade abdominal, diabetes, envelhecimento populacional e fatores bioquímicos como relação apolipoproteína B/A1, entre outros). Comportamentais: baixa escolaridade, tabagismo, falta de atividade física regular/sedentarismo, dieta inadequada, estresse e depressão, consumo moderado/elevado de bebida alcoólica) na ocorrência de DCV aterosclerótica;
- Analisar e discutir o perfil genômico associado à ocorrência de DCV aterosclerótica da população brasileira àquele de outras populações descrito em outros estudos já publicados.

3.3. Objetivo(s) do Plano Nacional de Saúde ao(s) qual(is) o projeto se vinculará:

Objetivo 03. Reduzir ou controlar a ocorrência de doenças e agravos passíveis de prevenção e controle. *Objetivo 04.* Fomentar a produção do conhecimento científico, promovendo o acesso da população às tecnologias em saúde de forma equitativa, progressiva e sustentável.

4. POLÍTICA(S) PÚBLICA(S) VINCULADA(S):

1. Programa Nacional de Genômica e Saúde de Precisão - Genomas Brasil, Portaria 1.949, de4 agosto de 2020;
2. Política Nacional de Promoção da Saúde (PNPS), Portaria 2.446, de 11 de novembro de 2014, que tem como um de seus objetivos específicos estimular a pesquisa, a produção e a difusão de conhecimentos e de estratégias inovadoras no âmbito das ações de promoçãode saúde;
3. Política Nacional de Atenção Cardiovascular de Alta Complexidade, Anexo XXXI da Portaria de Consolidação 2, de 28 de setembro de 2017, que consolida as normas sobre as políticasnacionais de saúde do SUS;
4. Portaria 210, de 15 de junho de 2004, que define unidades de assistência em alta complexidade cardiovascular e os centros de referência em alta complexidade cardiovascular e suas aptidões e qualidades;

Considerando a execução do projeto em tempos de pandemia de COVID-19, existe um risco potencial quanto à dificuldade de recrutamento e logística. Entretanto, em contrapartida, esse projeto será inserido no contexto dos grupos que desenvolveram os estudos INTERHEART e INTERSTROKE (1,2), os quais possibilitam segurança e estrutura organizacional, com facilitação do contato com os centros participantes, e garantem eficiência logística para o estudo.

Além disso, pensando na complexidade do processo de sequenciamento, devido à apuração na análise e serviço de bioinformática, pode haver atrasos ou entraves no recebimento de laudos. Entretanto, considerando a estrutura colaborativa, teremos uma rede assertiva para coleta, envio, análise de amostras biológicas e emissão de laudo, que facilitará o andamento da pesquisa. No momento do envio desta proposta, 50 centros de pesquisa, com especialidade em cardiologia demostraram interesse em participar como centros deste estudo. Ressalta-se a presença de centros em todas as cinco regiões brasileiras.

4.1. O projeto possui desafio (s) pertinente (s) ao desenvolvimento do SUS:

Como principal causa de mortalidade na população, a DCV deve ser o foco de programas de diagnóstico/intervenção que visam ao mapeamento de fatores de risco com o intuito de se estabelecer medidas para o controle dos mesmos e, com isso, reduzir a mortalidade e o impacto da DCV na população. Os principais fatores de risco cardiovascular já conhecidos (tabagismo, diabetes, hipertensão arterial, obesidade, ansiedade e depressão, dieta inadequada, sedentarismo, etilismo e relação apolipoproteína B/A1 (ApoB/ApoA1) são todos fatores modificáveis, entretanto, um dos fatores não-modificáveis é o *background* genético. Alguns estudos demonstram polimorfismos associados ao aumento do risco de IAM e AVE, mesmo quando ajustados para os fatores de risco habituais. Nesse contexto, a detecção de polimorfismos associados à DCV poderia identificar pacientes onde os fatores de risco modificáveis possam ser triados/tratados de forma precoce visando à prevenção cardiovascular.

4.2. Quais os benefícios ao SUS:

Por meio dessa proposta, espera-se obter um diagnóstico da frequência e do perfil de polimorfismos associados ao aumento do risco de DCV na população brasileira. A partir disso, espera-se que sejam elaboradas intervenções direcionadas ao diagnóstico/tratamento precoce das condições modificáveis associadas à DCV. Desse modo, espera-se a redução da morbidade/mortalidade cardiovascular em pacientes com preditores genéticos, após eventual implementação de estratégias baseadas nos resultados desse estudo (implementação da evidência). Os resultados do projeto serão analisados, divulgados e publicados, colaborando para a disseminação do conhecimento científico e para a implementação de sistemas

semelhantes em diversas localidades do território nacional. De fato, esse projeto irá fornecer dados para a plataforma do Programa Nacional de Genômica e Saúde de Precisão - Genomas Brasil, contribuindo assim para o desenvolvimento de uma ampla base de dados em genômica sobre a população brasileira.

5. JUSTIFICATIVA E RELEVÂNCIA DO PROJETO PARA O SUS

As doenças cardiovasculares são responsáveis por mais de 300.000 mortes ao ano, representando a principal causa de morte no Brasil, seguidas de neoplasias, doenças respiratórias e diabetes. Juntas, as doenças crônicas não transmissíveis (DCNT) são responsáveis por aproximadamente 70% das causas de morte em ambos os sexos (6,7).

Atualmente sabe-se que sete em cada dez casos de DCV podem ser explicados por fatores de risco como hipertensão arterial, baixa escolaridade, tabagismo, dislipidemia, dieta inadequada, obesidade abdominal, falta de atividade física regular, diabetes, e alterações psicossociais- todos os quais passíveis de serem modificados (8). Entretanto, nos últimos anos, o avanço nas tecnologias de sequenciamento genético associado à maior acessibilidade aos mesmos pela redução de custos, propiciou a identificação de polimorfismos genéticos associados ao maior risco de doenças cardiovasculares, reforçando o modelo de interação genética e meio ambiente como deflagrador das doenças cardiovasculares sendo, diferente dos outros fatores de risco conhecidos, fator não modificável (9–11).

6.1. Fatores de Risco

Estima-se que cerca de 90% dos casos de IAM e de AVE estejam associados a fatores de risco tradicionais, sendo que o envelhecimento populacional, o tabagismo, a dieta inadequada e o sedentarismo estão associados à crescente prevalência destes fatores. No âmbito nacional, temos limitações de dados representativos em termos de vigilância epidemiológica para DCNT e fatores de risco determinantes (6,8,12). O INTERHEART, estudo caso-controle que avaliou os efeitos dos fatores de risco potencialmente modificáveis associados ao IAM em 52 países e que envolveu 30000 indivíduos (15152 casos e 14820 controles) identificouque tabagismo, diabetes, hipertensão arterial, obesidade estresse e depressão, dieta inadequada, sedentarismo, etilismo e relação apolipoproteína B/A1 (ApoB/ApoA1) constituem os fatores de risco mais importantes associados ao primeiro IAM em todas as regiões geográficas, em homens e mulheres e com diversidade étnica representativa (2).

Na América Latina, a análise dos resultados foi coincidente com os observados pelo INTERHEART global, demonstrando especial importância para obesidade abdominal, dislipidemia, tabagismo e hipertensão. Tais

• Rua Treze de Maio, 1.815 • Paulista
• CEP 01327 001 • São Paulo • SP
• Fone: 11 3549 1976 • Fax: 11 3549 0394

resultados foram coincidentes com estudo previamente publicado, onde foram avaliados fatores de risco apenas na população brasileira (12–14).

O tabagismo está associado a diversas doenças e é responsável por 50% das mortes evitáveis em tabagistas, sendo a metade por DCV. Estima-se que metade das mortes por tabagismo ocorra em indivíduos entre 35-69 anos, os quais perdem em média 22 anos de vida devido ao hábito (13,15). No Brasil, a prevalência encontra-se ainda em 15,5%. Em todos os países, o hábito de fumar é mais prevalente em homens, entretanto a diferença desta proporção vem diminuindo e representa importante fator de risco em mulheres, com relação linear conforme o número de cigarros/dia (15).

A prevalência de diabetes em adultos vem aumentando progressivamente, com substancial contribuição das mudanças na dieta e obesidade. A mortalidade por gênero e idade é 58% maior nos indivíduos com diabetes em relação a controles não diabéticos. De todas as mortes em pacientes diabéticos, 38% são decorrentes de DCV (12,13,16,17). A presença de Diabetes eleva o risco cardiovascular em 2-3 vezes. O INTERHEART, por exemplo, revelou 4,2 vezes mais chances para ocorrência de IAM em diabéticos na população brasileira. Existe ainda um risco maior de IAM em mulheres com diabetes (2). Valores de hemoglobina glicada (HBA1C) > 5,4% estão associados a um risco cardiovascular 22% maior em relação aos indivíduos com valores mais baixos.

A hipertensão arterial (HAS) consiste em fator independente e mais prevalente para o risco cardiovascular. Estima-se que a prevalência de HAS na América Latina seja de 29,1% e no Brasil de aproximadamente 24%. Quando avaliados indivíduos acima de 60 anos, tal prevalência sobe para aproximadamente 50%. Em relação à DAC, o tratamento com anti-hipertensivos reduz o risco em 20-25% (18), entretanto, publicações descrevem taxas de controle da hipertensão arterial no Brasil entre 10,1% e 52,4%. Considerando-se uma estimativa otimista, cerca de 50% dos hipertensos tratados estariam expostos às complicações da doença pelo descontrole dos níveis pressóricos (18,19).

A elevação do LDL colesterol é um dos fatores mais importantes para DAC, porém seus valores são influenciados pela fase aguda do IAM ou ausência de jejum adequado para mensuração de seus níveis. A ApoB não é influenciada pelo nível de jejum e reflete a quantidade de lipoproteínas potencialmente aterogênicas, e a ApoA1 representa as lipoproteínas antiaterogênicas de alta densidade. Alguns estudos demonstram que a relação ApoB/ApoA1 é melhor preditor de IAM do que a relação LDL/HDL como foi observado no estudo AMORIS (2,20). Estima-se que 25% da população brasileira apresenta colesterol total acima de 190mg/dl, sendo que nos indivíduos maiores de 45 anos esta proporção sobe para 33%.

A obesidade, morbidade definida como valores de Índice de Massa Corporal (IMC) acima de 30kg/m^2,apresenta crescente expansão em sua prevalência, também se comportando como fator de risco independente para IAM (10). Atualmente, estima-se a proporção de 14,8% de obesos na população acima de 20 anos. A obesidade é também um reflexo de uma dieta inadequada e sedentarismo, hábitos cada vez mais

• Rua Treze de Maio, 1.815 • Paulista
• CEP 01327 001 • São Paulo • SP
• Fone: 11 3549 1976 • Fax: 11 3549 0394

frequentes no Brasil e globalmente. O estudo INTERHEART na América Latina demonstrou que uma relação cintura/quadril elevada está associada à chance de IAM 2,5 vezes maior nos homens e 4,1 vezes em mulheres (13,14). O INTERHEART América Latina também comprovou nítida associação inversa entre consumo de dieta rica em frutas e vegetais e ocorrência de IAM. Foi observada ainda associação positiva entre consumo de frituras e petiscos salgados e ocorrência de IAM (14,21). Com relação ao sedentarismo, dados demonstram que apenas 14,7% da população brasileira pratica atividades físicas regulares, sendo que apenas 3,3% realizam o mínimo de 30 minutos/dia, cinco vezes/semana recomendados pela OMS (14,17). A realização de atividades físicas regulares pode ser responsável por uma redução de 40% do risco relativo para ocorrência do primeiro IAM, porém, a proporção de indivíduos fisicamente ativos nesta população ainda é muito baixa (2).

Em 2010, o consumo de álcool foi responsável por 2,5 milhões de mortes no mundo principalmente devido às causas externas, cirrose hepática, DCV e câncer. Adicionalmente, a frequência e o padrão de consumo pode ser um importante modificador da associação entre consumo de álcool e IAM. Existem evidências que o consumo episódico de altas quantidades de álcool é um fator de risco para morte súbita cardíaca (22,23). No Brasil, apesar de 45% dos indivíduos serem abstêmios, a proporção de consumidores excessivos é de 25%.

No Brasil, estima-se que 18,8% dos indivíduos já receberam diagnóstico de depressão e, quando o critério de avaliação contempla transtornos depressivos e de ansiedade, este número alcança 30% (14). O estudo INTERHEART demonstrou que o efeito do estresse é independente do perfil socioeconômico e do tabagismo e consistente nas regiões geográficas, idade e gênero avaliados, e de modo independente após ajuste para covariáveis (24). Na América Latina, foi observada apenas discreta associação entre depressão e risco de IAM. No Brasil, foi observada uma razão de chances de 1,48 (IC 95% 1.0-2.22).

6.2. Fatores Genéticos e Doença Cardiovascular

No estudo da contribuição do *background* genético na patogênese de doenças, as principais formas de apresentação incluem a mutação em genes com potencial efeito direto de manifestação de doença (doenças monogênicas) ou genes que apresentam polimorfismos/mutações (doenças poligênicas) que se comportam como fatores de susceptibilidade para uma doença, no modelo patogênico de “two hits”, onde há necessidade de *background* genético associado a fatores ambientais para o desenvolvimento da doença (25–27).

Alguns estudos avaliaram o potencial impacto de polimorfismos na DCV. Uma sub-análise do estudo INTERHEART, o INTERHEART *Genetics Study*, num modelo de estudo caso-controle avaliou o impacto de 1536 *single-nucleotide polymorphisms* (SNPs) em 103 genes numa população multiétnica de 8034 pacientes. O objetivo foi avaliar o impacto de polimorfismos genéticos associados a DCV com o risco de IAM. Quando avaliados conjuntamente aos outros fatores de risco cardiovascular (APO B/A, hipertensão, diabetes, obesidade abdominal, tabagismo, sedentarismo, alcoolismo e depressão), treze polimorfismos foram

• Rua Treze de Maio, 1.815 • Paulista
• CEP 01327 001 • São Paulo • SP
• Fone: 11 3549 1976 • Fax: 11 3549 0394

associados a maior risco cardiovascular, sendo 11 desses relativos aos níveis séricos de APO B/A, 1 associado ao receptor de LDL e 1 à Apolipoproteína E (28). O estudo INTERHEART mostrou que os marcadores genéticos de risco para ocorrência de IAM, identificados por meio de análise do genoma, parecem estar amplamente associados ao IAM em vários grupos étnicos distintos. No entanto, o risco atribuível populacional (RAP) a esses fatores genéticos é relativamente pequeno em comparação aos fatores de risco modificáveis. Para o subgrupo de latino-americanos, o escore de risco genético foi (RAP 0,86 IC95% 0,74-0,93 e RAP 0,12 IC95% 0,02-0,42, respectivamente para fatores modificáveis e não modificáveis). Entretanto, como já mencionado, não há dados exclusivamente de indivíduos brasileiros com poder estatístico adequado e com sistematização da obtenção de informações sobre fatores de risco tradicionais, aspectos essenciais e, desse modo, recomendáveis para uma análise integrada com objetivo de avaliar a contribuição do escore de risco genético sobre o impacto no risco atribuível populacional para a ocorrência de eventos ateroscleróticos em seu amplo espectro clínico.

A população brasileira, em função de sua diversidade étnica, miscigenação, múltiplas ancestralidades e alta prevalência de casamentos consanguíneos possui peculiaridade genética ímpar e que pode refletir na predisposição a doenças, dentre elas, as cardiovasculares (29,30). A interferência do componente genético sobre o padrão ou suscetibilidade a doenças na população brasileira já demonstrou ter impacto sobre o controle e tratamento de doenças infecciosas, autoimunes, hematológicas, farmacocinética de drogas e até sobre o sistema de alocação de transplantes (31–37). Em um artigo de revisão sobre saúde cardiovascular no Brasil (38), são descritos diversos pontos sobre questões importantes inerentes ao sistema público de saúde e características epidemiológicas, além dos fatores já conhecidos associados à DCV. Os autores pontuam os próximos desafios para uma melhor compreensão sobre a DCV no Brasil e citam, dentre eles, a necessidade de estudos robustos, com representatividade nacional, que permitam o conhecimento das peculiaridades epidemiológicas e contribuintes genéticos para a gênese da DCV. Desse modo, propomos responder a esta questão populacional relevante por meio do Estudo CV-Genes, com correção/ajuste pelos fatores de risco cardiovascular tradicionais, visto que o *background* genético é o único fator de risco não controlável ou modificável e conhecer seu real impacto sobre a população brasileira pode suscitar a realização de programas de *screening* genético-populacionais para controle estrito de fatores de risco cardiovascular e/ou indicar mudanças em alvos terapêuticos e de controle clínico.

Recentemente foi publicada uma revisão (39) dos principais estudos que avaliaram loci de susceptibilidade para doença arterial coronariana. Tal revisão englobou estudos de consórcios importantes como o CARDIoGRAM, MIGen, WTCCC and Cardiogenics, deCODE, CARDIoGRAM, C4D e CARDIoGRAM + C4D que, em conjunto, contribuíram com a identificação de 60 loci, cujo mecanismo de ação se relaciona aos níveis séricos de LDL, lipoproteína A e triglicerídeos, pressão arterial, índice de massa corporal, perfil de coagulação, alterações nas células endoteliais e musculares lisas da parede vascular, mecanismos de migração e adesão

• Rua Treze de Maio, 1.815 • Paulista
• CEP 01327 001 • São Paulo • SP
• Fone: 11 3549 1976 • Fax: 11 3549 0394

celular, ativação imunológica, inflamação, crescimento celular, diferenciação e apoptose, assim como constituintes da matriz extracelular e também alguns loci com função desconhecida. A tabela 1, extraída do artigo em questão, elenca os potenciais loci, potenciais mecanismos de ação e de qual consórcio tal informação foi adquirida.

Polimorfismos em genes associados à regulação dos níveis de cálcio (*CASR, CYP24A1, CARS, DGKD, DGKH/KIAA0564 e GATA3*) (40) e metaloproteinases (MMP-3 e MMP-9) (41) também foram associados ao aumento do risco de doença arterial coronariana e IAM. A lista de polimorfismos associados ao IAM aumenta progressivamente, e alguns autores já reforçam a importância do uso do *background* genético em calculadoras de risco cardiovascular (9,42,43).

Em relação aos casos de Acidente Vascular Cerebral (AVC), polimorfismos em alguns genes como *MTHFR, eNOS, ACE, AGT, ApoE, PON1, PDE4D* foram associados a um maior risco de AVC isquêmico. De forma esperada, polimorfismos em alguns genes. Para os casos de AVC hemorrágico, polimorfismos em genes do colágeno (44,45), TLR4 e CD14 (46) e até do gene que dá origem à proteína C-reativa foram identificados (47). A tabela 2 e a Figura 2 trazem o resumo de polimorfismos associados ao aumento do risco de AVC isquêmico e hemorrágico.

• Rua Treze de Maio, 1.815 • Paulista
• CEP 01327 001 • São Paulo • SP
• Fone: 11 3549 1976 • Fax: 11 3549 0394

Tabela 1. Resumo dos *loci* de susceptibilidade à doença arterial coronariana (extraído de Assimes et al[29], Genetics: implications for preventionand management of coronary artery disease. JACC, 2016)

Lead SNP†	Chromosome	Nearest Genes‡	Frequency of Allele Raising Risk	OR (95% CI)	p Value§	Potential Mechanism of Action‖	Year Locus First Reported to Reach Genome-Wide Significance	Consortium and/or Author (Ref. #)
rs11206510	1	*PCSK9*	0.85	1.08 (1.05-1.11)	2.340×10^{-8}	LDL levels	2009	MIGen (59)
rs7528419	1	*SORT1*	0.79	1.12 (1.10-1.15)	1.970×10^{-23}	LDL levels	2007	WTCCC and Cardiogenics (56)
rs515135	2	*APOB*	0.79	1.07 (1.04-1.10)	3.090×10^{-8}	LDL levels	2013	CARDIoGRAM+C4D (63)
rs6544713	2	*ABCG5-ABCG8*	0.32	1.05 (1.03-1.07)	8.880×10^{-7}	LDL levels	2013	CARDIoGRAM+C4D (63)
rs56289821	19	*LDLR*	0.90	1.14 (1.11-1.18)	4.440×10^{-15}	LDL levels	2009	MIGen (59)
rs4420638	19	*APOE-APOC1*	0.17	1.10 (1.07-1.13)	7.070×10^{-11}	LDL levels	2013	CARDIoGRAM+C4D (63)
rs3184504	12	*SH2B3*	0.42	1.07 (1.04-1.09)	1.030×10^{-9}	LDL levels, BP	2009	deCODE (58)
rs55730499	6	*SLC22A3-LPAL2-LPA*	0.06	1.37 (1.31-1.44)	5.390×10^{-39}	Lp(a) levels	2009	WTCCC and Cardiogenics (60)
rs264	8	*LPL*	0.85	1.06 (1.03-1.09)	1.060×10^{-5}	TRIG levels	2013	CARDIoGRAM+C4D (63)
rs2954029	8	*TRIB1*	0.55	1.04 (1.03-1.06)	2.610×10^{-6}	TRIG levels	2013	CARDIoGRAM+C4D (63)
rs964184	11	*ZNF259-APOA5-APOA1*	0.18	1.05 (1.03-1.08)	5.600×10^{-5}	TRIG levels	2011	CARDIoGRAM (62)
rs17609940	6	*ANKS1A*	0.82	1.03 (1.00-1.05)	3.000×10^{-2}	HDL levels, height	2011	CARDIoGRAM (62)
rs3918226	7	*NOS3*	0.06	1.14 (1.09-1.19)	1.690×10^{-9}	BP	2015	1GP CARDIoGRAM+C4D (54)
rs2681472	12	*ATP2B1*	0.20	1.08 (1.05-1.10)	6.170×10^{-11}	BP	2012	Lu et al. (64)
rs17514846	15	*FURIN-FES*	0.44	1.05 (1.03-1.07)	3.100×10^{-7}	BP	2013	CARDIoGRAM+C4D (63)
rs72689147	4	*GUCY1A3*	0.82	1.07 (1.05-1.10)	6.070×10^{-9}	BP, cell growth/differentiation/ apoptosis	2013	CARDIoGRAM+C4D (63)
rs11830157	12	*KSR2*	0.36	1.12 (1.08-1.16)¶	2.120×10^{-9}	BMI	2015	1GP CARDIoGRAM+C4D (54)
rs663129	18	*PMAIP1-MC4R*	0.26	1.06 (1.04-1.08)	3.200×10^{-8}	BMI	2015	1GP CARDIoGRAM+C4D (54)
rs4252185	6	*PLG*	0.06	1.34 (1.28-1.41)	1.640×10^{-32}	coagulation	2013	CARDIoGRAM+C4D (63)
rs2519093	9	*ABO*	0.19	1.08 (1.06-1.11)	1.190×10^{-11}	coagulation, LDL levels	2011	CARDIoGRAM (62)
rs9349379	6	*PHACTR1*	0.43	1.14 (1.12-1.16)	1.810×10^{-42}	Arterial vessel wall endothelial cell	2009	MIGen (59)
rs9319428	13	*FLT1*	0.31	1.04 (1.02-1.06)	7.130×10^{-5}	Arterial vessel wall endothelial cell	2013	CARDIoGRAM+C4D (63)
rs8042271	15	*MFGE8-ABHD2*	0.90	1.10 (1.06-1.14)	3.680×10^{-8}	Arterial vessel wall endothelial cell	2015	1GP CARDIoGRAM+C4D (54)
rs7212798	17	*BCAS3*	0.15	1.08 (1.05-1.11)	1.880×10^{-8}	Arterial vessel wall endothelial cell	2015	1GP CARDIoGRAM+C4D (54)
rs4593108	4	*EDNRA*	0.80	1.07 (1.05-1.10)	8.820×10^{-10}	Arterial vessel wall smooth muscle cell	2013	CARDIoGRAM+C4D (63)
rs17087335	4	*REST-NOA1*	0.21	1.06 (1.04-1.09)	4.590×10^{-8}	Arterial vessel wall smooth muscle cell	2015	1GP CARDIoGRAM+C4D (54)
rs12202017	6	*TCF21*	0.70	1.07 (1.05-1.09)	1.980×10^{-11}	Arterial vessel wall smooth muscle cell	2011	CARDIoGRAM (62)
rs2107595	7	*HDAC9*	0.20	1.08 (1.05-1.10)	8.050×10^{-11}	Arterial vessel wall smooth muscle cell	2013	CARDIoGRAM+C4D (63)
rs2891168	9	*CDKN2BAS (9p21)*	0.49	1.21 (1.19-1.24)	2.290×10^{-98}	Arterial vessel wall smooth muscle cell	2007	McPherson et al., deCODE, WTCCC (46-48)
rs11191416	10	*CYP17A1-CNNM2-NT5C2*	0.87	1.08 (1.05-1.11)	4.650×10^{-9}	Arterial vessel wall smooth muscle cell	2011	CARDIoGRAM (62)
rs4468572	15	*ADAMTS7*	0.59	1.08 (1.06-1.10)	4.440×10^{-16}	Arterial vessel wall smooth muscle cell	2011	C4D, Reilly, CARDIoGRAM (61,62,89)
rs10840293	11	*SWAP70*	0.55	1.06 (1.04-1.08)	1.280×10^{-8}	Arterial vessel wall smooth muscle cell, inflammation/immune system/ cell migration-adhesion	2015	1GP CARDIoGRAM+C4D (54)
rs17678683	2	*ZEB2-AC074093.1*	0.09	1.10 (1.07-1.14)	3.000×10^{-9}	Cell growth/differentiation/apoptosis	2013	CARDIoGRAM+C4D (63)
rs2128739	11	*PDGFD*	0.32	1.07 (1.05-1.09)	7.050×10^{-11}	Cell growth/differentiation/apoptosis	2011	C4D (61)
rs56062135	15	*SMAD3*	0.79	1.07 (1.05-1.10)	4.520×10^{-9}	Cell growth/differentiation/apoptosis	2015	1GP CARDIoGRAM+C4D (54)
rs46522	17	*UBE2Z*	0.51	1.04 (1.02-1.06)	1.840×10^{-5}	Cell growth/differentiation/apoptosis	2011	CARDIoGRAM (62)
rs9970807	1	*PPAP2B*	0.92	1.13 (1.10-1.17)	5.000×10^{-14}	Inflammation/immune system/cell migration-adhesion	2011	CARDIoGRAM (62)
rs2487928	10	*KIAA1462*	0.42	1.06 (1.04-1.08)	4.410×10^{-11}	Inflammation/immune system/cell migration-adhesion	2011	C4D (61)
rs1870634	10	*CXCL12*	0.64	1.08 (1.06-1.10)	5.550×10^{-15}	Inflammation/immune system/cell migration-adhesion	2007	WTCCC and Cardiogenics (56)
rs1412444	10	*LIPA*	0.37	1.07 (1.05-1.09)	5.150×10^{-12}	Inflammation/immune system/cell migration-adhesion	2011	C4D (61)
rs6689306	1	*IL6R*	0.45	1.06 (1.04-1.08)	2.600×10^{-9}	Inflammation/immune system/cell migration-adhesion, cell growth/ differentiation/apoptosis	2013	CARDIoGRAM+C4D (63)
rs67180937	1	*MIA3*	0.66	1.08 (1.06-1.11)	1.010×10^{-12}	Extracellular matrix	2007	WTCCC & Cardiogenics (56)
rs11838776	13	*COL4A1/A2*	0.26	1.07 (1.05-1.09)	1.830×10^{-10}	Extracellular matrix	2011	CARDIoGRAM (62)
rs16986953	2	*AK097927*	0.10	1.09 (1.06-1.12)	1.450×10^{-8}	Other/unknown	2013	CARDIoGRAM+C4D (63)
rs7568458	2	*VAMP5-VAMP8-GGCX*	0.45	1.06 (1.04-1.08)	3.620×10^{-10}	Other/unknown	2013	CARDIoGRAM+C4D (63)
rs6725887	2	*WDR12*	0.11	1.14 (1.11-1.18)	9.510×10^{-18}	Other/unknown	2009	MIGen (59)
rs9818870	3	*MRAS*	0.14	1.07 (1.04-1.10)	2.210×10^{-6}	Other/unknown	2009	Cardiogenics (57)
rs273909	5	*SLC22A4-SLC22A5*	0.12	1.06 (1.03-1.09)	1.240E−04	Other/unknown	2013	CARDIoGRAM+C4D (63)
rs6903956	6	*ADTRP-C6orf105*	0.35	1.00 (0.98-1.02)	9.600×10^{-1}	Other/unknown	2011	Wang et al. (176)
rs56336142	6	*KCNK5*	0.81	1.07 (1.04-1.09)	1.850×10^{-8}	Other/unknown	2013	CARDIoGRAM+C4D (63)
rs10953541	7	*7q22*	0.78	1.05 (1.03-1.08)	1.020×10^{-5}	Other/unknown	2011	C4D (61)
rs11556924	7	*ZC3HC1*	0.69	1.08 (1.05-1.10)	5.340×10^{-11}	Other/unknown	2011	CARDIoGRAM (62)
rs10139550	14	*HHIPL1*	0.42	1.06 (1.04-1.08)	1.380×10^{-8}	Other/unknown	2011	CARDIoGRAM (62)
rs216172	17	*SMG6*	0.35	1.05 (1.03-1.07)	5.070×10^{-7}	Other/unknown	2011	CARDIoGRAM (62)
rs12936587	17	*RAI1-PEMT-RASD1*	0.61	1.03 (1.01-1.05)	8.240×10^{-4}	Other/unknown	2011	CARDIoGRAM (62)
rs12976411	19	*ZNF507-LOC400684*	0.91	1.49 (1.38-1.67)¶	1.180×10^{-14}	Other/unknown	2015	1GP CARDIoGRAM+C4D (54)
rs28451064	21	*KCNE2* (gene desert)	0.12	1.14 (1.10-1.17)	1.330×10^{-15}	Other/unknown	2009	MIGen (59)
rs180803	22	*POM121L9P-ADORA2A*	0.97	1.20 (1.13-1.27)	1.640×10^{-10}	Other/unknown	2015	1GP CARDIoGRAM+C4D (54)

• Rua Treze de Maio, 1.815 • Paulista
• CEP 01327 001 • São Paulo • SP
• Fone: 11 3549 1976 • Fax: 11 3549 0394

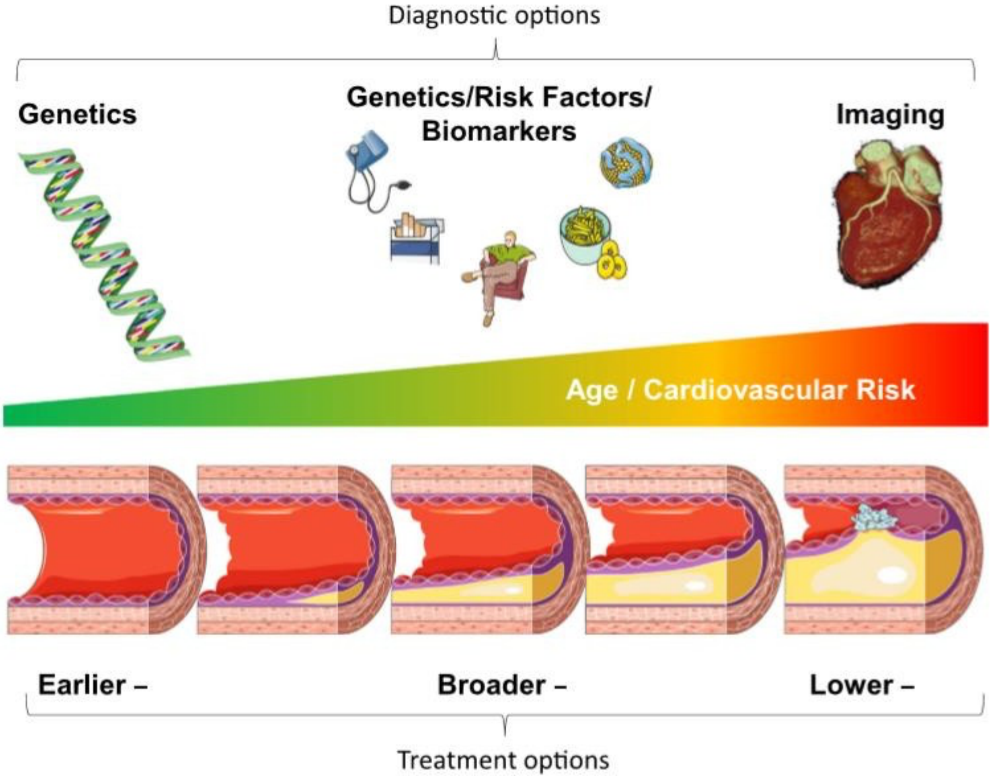

Figura 1. Associação de fatores genéticos e comportamentais com estratégias de diagnóstico de doença cardiovascular (extraído de Lechner et al (42))

Tabela 2. *Loci* de risco para AVC isquêmico e hemorrágico (extraído de Chauhan et al (48))

Locus	Lead-SNP	Chromosome	Position	Phenotype	Risk Allele	Risk Allele Frequency	N[a]	OR	P
Ischemic stroke									
TSPAN2 [51•]	rs12122341	1	115655690	LAS	G	0.25	20,941/364,736	1.19	1.30×10^{-9}
PITX2 [29]	rs6843082	4	111718067	CE	G	0.21	2365/12,389	1.36	7.8×10^{-16}
FOXF2 [39•]	rs12204590	6	1337393	AS, *SVD*[‡]	A	0.21	24,164/155,765	1.08	1.48×10^{-8}
CDC5L [32]	rs556621	6	44594159	LAS	A	0.33	400/1172	1.62	3.9×10^{-8}
HDAC9 [29]	rs2107595	7	19049388	LAS	A	0.16	2167/12,389	1.39	2.0×10^{-16}
ABO [40•]	rs505922	9	136149229	LAS, IS	A	0.19	26,127/53,788	1.09	4.3×10^{-8}
HABP2 [41•]	rs11196288	10	115057443	IS	G	0.05	5508/29,713	1.41	9.5×10^{-9}
MMP12 [28•]	rs660599	11	102729757	LAS	A	0.19	3197/62,912	1.18	2.6×10^{-8}
NINJ2 [34]	rs11833579	12	775199	IS	A	0.23	1164/18,058	1.41	2.3×10^{-10}
SH2B3/ALDH2 [33•]	rs10744777	12	112233018	IS, SVD	T	0.66	17970/70,764	1.1	7.1×10^{-11}
PRKCH [59]	rs2230500	14	61924239	SVD	A	0.19	2246/2971	1.4	5.1×10^{-7}
AQP9 [42•]	rs4471613	15	58551694	AS	A	0.02	1592/13,153	2.27	3.9×10^{-8}
ZFHX3 [29]	rs879324	16	73068678	CE	A	0.19	2365/12,389	1.25	2.3×10^{-8}
Intracerebral hemorrhage									
PMF1 [30•]	rs2984613	1	156197380	ICH (deep)	C	0.32	881/1481	1.33	2.2×10^{-10}
APOE [38]	rs429358	19	45411941	ICH (lobar)	ε2	0.07	931/3744	1.82	6.6×10^{-10}
APOE [38]	rs429358	19	45411941	ICH (lobar, *deep*[b])	ε4	0.12	931/3744	2.2	2.4×10^{-11}

• Rua Treze de Maio, 1.815 • Paulista
• CEP 01327 001 • São Paulo • SP
• Fone: 11 3549 1976 • Fax: 11 3549 0394

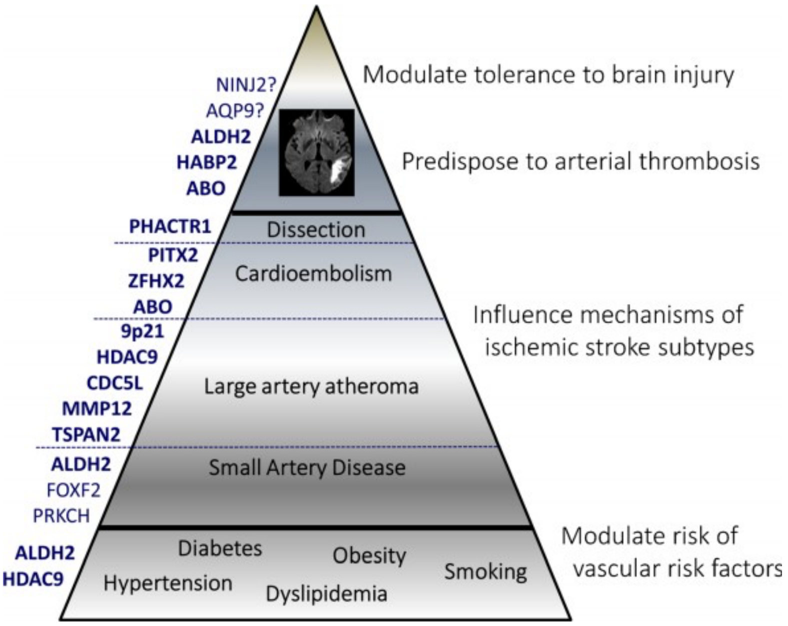

Figura 2. Fatores de risco para AVC isquêmico em vários níveis, com genes associados à fisiopatogênese (extraído de Chauhan et al (48))

6.3. Contribuições esperadas

O primeiro ponto é agregar pacientes à base de dados do projeto Genomas Brasil e contribuir para que esta seja representativa e heterogênea o suficiente para abranger os mais variados estratos populacionais e, especificamente, para aqueles estratos patogênicos, agregar informações de condições extensivamente influenciadas de forma poligênica. Segundo, será possível avaliar o uso de técnica mais moderna, menos onerosa e efetiva (WGS acoplado a exoma), para a detecção de polimorfismos genéticos e cálculo de *Polygenic Risk Score*. Terceiro, doenças cardiovasculares representam um grande impacto em saúde pública, seja em custo ou morbimortalidade. A partir desse estudo, poderá ser desenvolvido um painel genético/clínico de diagnóstico para a prevenção de eventos cardiovasculares agudos que, mesmo a despeito da pequena fração de risco genético esperada, trará uma informação corrigida por diversos fatores modificáveis. Ressalta-se que o desenvolvimento de tecnologias é permitido com base em minuta do Projeto Genomas Brasil. Quarto, teremos o perfil genético e o seu risco atribuível exclusivamente para a população brasileira, e isso será um resultado sem precedentes, seja para corroborar com achados de estudos que incluíram população latino-americana, como o INTERHEART, ou para trazer novas conclusões.

Estudos mostraram que o *background* genético pode exercer efeito significativo para o aumento do risco de DCV. Em população miscigenada como a nossa, determinar o risco atribuível a esta condição poderá propiciar o desenvolvimento de ações direcionadas a subpopulações específicas, no intuito de aconselhar

• Rua Treze de Maio, 1.815 • Paulista
• CEP 01327 001 • São Paulo • SP
• Fone: 11 3549 1976 • Fax: 11 3549 0394

geneticamente e proporcionar ações de monitoramento e prevenção. Isso é particularmente importante considerando que alterações de estilo de vida e mesmo a correta adesão a terapias farmacológicas não sejam implementadas ou seguidas de forma sustentada. Consideramos esse um passo importantíssimo, juntamente com outras iniciativas, para a otimização de uma medicina de precisão, com base genética, capitaneada pelo Projeto Genomas Brasil.

6.4. Custo-Efetividade da metodologia a ser empregada

Atualmente existem diversas publicações avaliando a custo-efetividade do uso combinado da conduta clínica associada ao monitoramento genético, seja para condições cardiovasculares com influência monogênica(49–51) ou poligênica(52–54). Ademais, é sempre importante considerar que o PRS possui pontos positivos e negativos e que, quando utilizado com responsabilidade e boa aplicabilidade, também têm o potencial de melhorar o acesso equitativo aos cuidados preventivos, servindo como um modelo que se alinha e estimula outras iniciativas de equidade na medicina(55).

Essas publicações mostram que a alternativa combinada é custo-efetiva. Portanto, o resultado da nossa proposta poderá ser utilizado para a condução de estudos de custo-efetividade, e assim, ter uma modelagem adaptada para a realidade brasileira, conferindo grande validade interna. Considerando instâncias de incorporação como a CONITEC, nossos dados poderão subsidiar a tomada de decisão, utilizando de *Real-World Evidence* derivada da nossa realidade, acoplada a dados de custo em moeda corrente. Com certeza, é o tipo de estudo que poderá ser conduzido após a conclusão das análises propostas no presente plano de trabalho.

6. EXPERTISE DO HOSPITAL DE EXCELÊNCIA PARA EXECUTAR O PROJETO

O Hospital Alemão Oswaldo Cruz, como entidade de excelência, atua de maneira ampliada em diversas especialidades, entre elas a cardiologia, a endocrinologia/metabologia e a neurologia, especialidades essas relacionadas e responsáveis pela prevenção, cuidado e monitoramento das DCV.

Além disso, o Hospital Alemão Oswaldo Cruz, por meio de seu Instituto Social, administra o Complexo Hospital dos Estivadores em Santos (SP), um hospital público, que traz os desafios e práticas cotidianas relacionadas à organização do Sistema Único de Saúde (SUS), propiciando um cenário real para o desenvolvimento de ações de pesquisa no contexto da APS e, também, da atenção especializada.

De fato, o Centro Internacional de Pesquisa (CIP-HAOC) apresenta como recursos humanos um grupo de trabalho composto por pesquisadores e profissionais de pesquisa clínica com extensa formação em epidemiologia, bioestatística, gerenciamento de dados, condução de estudos clínicos e estudos populacionais multicêntricos nacionais e internacionais e, de forma recente, análise genética e correção genótipo/fenótipo.

• Rua Treze de Maio, 1.815 • Paulista
• CEP 01327 001 • São Paulo • SP
• Fone: 11 3549 1976 • Fax: 11 3549 0394

Tal grupo de trabalho engloba profissionais de saúde com formações diversas, cientistas de dados, epidemiologistas, monitores de pesquisa, bibliotecária, entre outros. É importante ressaltar que, no contexto das DCV, o CIP-HAOC participa e já participou de relevantes estudos clínicos, tais como o de coorte populacional (PURE) e de Insuficiência Cardíaca (G-CHF), contribuindo para perspectivas atuais sobre tratamentos, monitoramento, definição de fatores de risco e prevenção. Recentemente, inicia também participação de estudos em genética e fatores de risco para doenças na área de Nefrologia, com resultados ainda a serem publicados.

O Hospital alemão Oswaldo Cruz também é protagonista no PROADI-SUS já no quinto triênio deste programa, com o desenvolvimento de ações direcionadas para a atenção primária, seja nas medidas de controle de monitoramento, na estruturação da atenção Farmacêutica, na identificação de oportunidades de intervenção para a melhoria do serviço e agora, por meio desta proposta, na avaliação de *background* genético como fatores de risco para doenças cardiovasculares.

O Hospital Alemão Oswaldo Cruz conta com um Centro Internacional de Pesquisa (CIP), que é uma unidade de pesquisa avançada, com foco em pesquisa clínica, epidemiológica, saúde populacional e avaliação de tecnologias em saúde. O CIP é composto por pesquisadores renomados e que já participaram de grandes estudos na área de cardiologia, mas também estudos que associam a avaliação genética acoplada à cardiologia, que é o caso desta presente proposta. Esta unidade tem extensiva interação com o Population Health Research Instituite (PHRI), da Universidade McMaster, no Canadá, e alguns de seus pesquisadores médicos, Álvaro Avezum (http://lattes.cnpq.br/5000252539139347) e Gustavo Oliveira (http://lattes.cnpq.br/6396650558105106), participaram de importantes iniciativas na área de cardiologia, como os estudos COMPASS, INTERHEART, INTERSTROKE e a maior coorte ativa no mundo atualmente, o Prospective Urban and Rural Epidemiological study (PURE).

Além disso, esta proposta terá como pesquisador médico Precil Diego Miranda de Menezes Neves (http://lattes.cnpq.br/0327186716740813), médico nefrologista com extensa atuação na área de genética, inclusive em instâncias de interface com o grande projeto global, o Human Genome Project.

O HAOC conta com estrutura de Biobanco código: B-108, aprovado pelo parecer Nº 27/2021-CONEP/SECNS/MS.

Ademais, o HAOC conta com estrutura de bioinformática necessária para a condução do estudo, por meio do centro de Inovação e Saúde Digital, sob a coordenação de Atualpa Carvalho de Aguiar (http://lattes.cnpq.br/0765461910923108) e um grupo de arquitetos de dados.

Na Sustentabilidade e Responsabilidade Social, a presente proposta será gerenciada por Haliton Oliveira (http://lattes.cnpq.br/2559137712702351), Gerente de Pesquisas da área, e com experiência na área de pesquisa clínica, epidemiológica, ATS, modelagem e análises econômicas.

Além da qualificação do HAOC, contaremos com a parceria do Laboratório Fleury S.A, o qual é uma das grandes potências em análises clínicas na América Latina e será responsável por realizar as análises genéticas planejadas na presente proposta. Diante das informações supracitadas e a organização para logística do estudo, acreditamos ter todas as competências necessárias para a boa condução do estudo proposto.
O projeto terá como pesquisador principal do HAOC: Álvaro Avezum Junior (http://lattes.cnpq.br/5000252539139347). A representante do laboratório Fleury será Maria Carolina Tostes Pintão (http://lattes.cnpq.br/2058570609326470)

7. METODOLOGIA

A descrição da metodologia deste estudo de caso-controle seguirá os itens estabelecidos pela ferramenta de relato *The Strengthening the Reporting of Observational Studies in Epidemiology (STROBE)* (56).

7.1. Delineamento do estudo

Trata-se de um estudo de caso-controle cardiovascular com incorporação de dados genéticos para avaliar o impacto da informação genômica, previamente associada às DCV poligênicas, como preditor de risco independente (expresso em *Odds Ratio*) e em conjunto com fatores de risco tradicionais (tabagismo, diabetes, hipertensão arterial, obesidade, ansiedade e depressão, dieta inadequada, sedentarismo, etilismo e relação apolipoproteína B/A1 (ApoB/ApoA1).

7.2. Contexto

O estudo será realizado em cerca de 50 centros participantes que prestem atendimento em saúde geral, incluindo para DCV, compreendendo as cinco regiões brasileiras. O estudo será conduzido de janeiro de 2022 até dezembro de 2023. A coleta de dados será realizada em cada centro de maneira consecutiva, para casos e controles, através de CRF eletrônica, durante o período de um ano e meio para a condução do estudo.
Os centros serão contatados e, caso aceitem participar, um contrato de pesquisa será firmado. Conforme rubrica específica detalhada no orçamento do projeto, tais centros receberão um valor pelo atendimento de cada paciente, considerando a necessidade de preenchimento correto de dados no e-CRF, garantia da qualidade de dados, coleta de material biológico, preparo pré-analítico e estocagem até transferência. Cada centro receberá um kit contendo agulhas, suporte, coletor, tubos, garrote, blood stop etc., necessários à coleta do material biológico. Os participantes serão recrutados durante a fase de hospitalização para manejo do evento aterotrombótico agudo (casos), mediante convite da equipe do centro participante, desde que

• Rua Treze de Maio, 1.815 • Paulista
• CEP 01327 001 • São Paulo • SP
• Fone: 11 3549 1976 • Fax: 11 3549 0394

atendam aos critérios definidos na subseção “participantes”, da seção de metodologia. Os controles serão pessoas com outras condições que não as DCV ou mesmo sem quaisquer condições clínicas, provenientes da mesma comunidade. Para participarem, os indivíduos casos e controles deverão assinar um TCLE fornecendo consentimento e ciência sobre os procedimentos da pesquisa, após serem informados por um pesquisador de cada equipe participante.

Será considerado inelegível para o estudo o participante que, porventura, estiver participando de qualquer outro projeto de pesquisa vinculado ao Programa Genomas Brasil, seja atrelado ao PROADI-SUS ou não. Essa informação consta no *Feasibility* do estudo e será largamente difundida no momento de treinamento dos centros de pesquisa. Essa atitude visa evitar duplicidades no banco de dados do projeto genomas Brasil.

Considerando a grande miscigenação da população brasileira e centros participantes nas cinco regiões brasileiras, acreditamos que o cálculo amostral será representativo da heterogeneidade genética da nossa população. Considerando que, de acordo com os dados do IBGE, a região sudeste concentra 42% da população brasileira, nordeste 27%, sul 14%, norte 9% e centro-oeste 8%, e que o número de casos previsto é de 1867, o N amostral por região será ajustado, preferencialmente, para 784 participantes de pesquisa na região Sudeste, 504 no Nordeste, 262 no Sul, 168 no Norte, e 149 na região Centro-Oeste. Para os controles será respeitada a mesma proporção. Do ponto de vista pragmático da pesquisa clínica, sabemos que essa uniformidade não acontecerá. Sendo assim, nos comprometemos com um extenso e qualificado processo de monitoria desses centros, para garantir a devida representatividade das regiões brasileiras.

Para manter uma priorização aos Centros de Referência que atendem ao SUS, assim como em outras iniciativas em pesquisa no âmbito do PROADI-SUS, a nossa proposta é estratificar em uma razão 70%/30% a proporção entre centros públicos e privados, respectivamente.

Todos os centros receberão treinamento quanto ao protocolo de pesquisa e boas práticas de pesquisa clínica. Todos os centros participantes prestam assistência em saúde incluindo na área de DCV. O recrutamento será feito por meio do contato com os centros tendo como referência um Pesquisador Principal em cada centro. Ao final do estudo, os centros receberão treinamento para a realização do aconselhamento genético para aqueles pacientes que apresentem variantes monogênicas associadas a um maior risco para doença cardiovascular, como em casos de hipercolesterolemia hereditária. Para o restante dos pacientes, será emitidoum relatório constando a contribuição do PRS ajustados pelos fatores de risco cardiovascular modificáveis, demonstrando o real impacto do componente genético como fator de risco cardiovascular não modificável. Do ponto de vista da assistência e acompanhamento clínico, todos os centros possuem estrutura necessária, validado após análise do questionário de factibilidade preenchido por cada centro. Conforme destacado no plano de trabalho, estamos tratando de uma condição poligênica e, possivelmente (com base em literatura jácitada acima), com uma discreta fração atribuível de risco de DCV pelo PRS em relação aos demais fatores de

• Rua Treze de Maio, 1.815 • Paulista
• CEP 01327 001 • São Paulo • SP
• Fone: 11 3549 1976 • Fax: 11 3549 0394

risco modificáveis (tradicionais). Nesse cenário, a comunicação com os pacientes inclui o resultado completo da análise ajustada, a qual é a proposta principal deste projeto de pesquisa. Dessa forma, a devida orientação e aconselhamento genético serão fornecidos aos pacientes pelos centros participantes, nos casos em que se detecte variantes monogênicas associadas a dislipidemias ou após devido ajuste do PRS pelos fatores fenotípicos, ou seja, após a conclusão e publicação do estudo.

7.3. Participantes

Os casos (N = 1867) serão selecionados pela ocorrência do primeiro evento cardiovascular aterosclerótico (Infarto Agudo do Miocárdio, Acidente Vascular Encefálico, e Evento Trombótico-Isquêmico Arterial Periférico) durante a fase de hospitalização para manejo do evento aterotrombótico agudo. A proporção entre casos e controles será de 1:1. Os controles (N = 1867) serão indivíduos adultos maiores de 18 anos que procuraram atendimento médico nos mesmos locais por outras questões clínicas (sem DCV) ou indivíduos sem qualquer doença manifesta. As definições dos eventos aterotrombóticos agudos listados acima seguem os critérios clínicos e de exames complementares clássicos e baseados em diretrizes nacionais e internacionais. O projeto completo será submetido ao sistema CEP/CONEP e, após aprovação ética, todos os casos e controles serão convidados a participar e, se concordarem, um Termo de Consentimento Livre e Esclarecido será obtido.

Os casos serão validados por comitê de adjudicação de eventos através de dados clínicos, exames complementares, sumário de hospitalização, dentre outros, usando metodologia utilizada e validada para diagnósticos (Estudo PURE e CID-10).

Faz parte do processo de qualificação da elegibilidade a um estudo caso controle que os casos sejamincidentes. Casos prevalentes podem carrear viés de memória e não refletir adequadamente a estimativa de “risco”, seja super ou subestimando o valor real, pois estes podem passar por influência de modificação de estilo de vida, da assistência à saúde, epidemias, alteração de políticas, entre outros. Além disso, as medidas de prevenção a serem implementadas visam não somente à ocorrência do primeiro evento, mas a todos os eventos subsequentes, obviamente porque os conceitos são os mesmos, ou seja, os fatores de risco independentes serão modificáveis para o evento primário assim como os demais. Não há premissa biológica ou científica para medidas de prevenção diferentes. O delineamento é mandatório para reduzir o impacto de todos os vieses e fatores confundidores, não restritos aos mencionados acima. Dessa forma, é por essa razão que selecionaremos casos incidentes e de primeira ocorrência.

Do ponto de vista de estudos caso-controle, já realizados para identificar os fatores de risco com Odds Ratio e RAP associados à ocorrência de IAM e AVE, nove (09) fatores de risco modificáveis foram identificados como independentemente associados para ambas as condições. Isto é suficiente, mais robusto e relevante do que o pensamento fisiopatológico ou análise mecanística isolados, até porque as medidas de prevenção devem ser

• Rua Treze de Maio, 1.815 • Paulista
• CEP 01327 001 • São Paulo • SP
• Fone: 11 3549 1976 • Fax: 11 3549 0394

estabelecidas com base em resultados de estudos clínicos, não com base em estudos de fisiopatologia, ou modelos animais, ou *in vitro*. De fato, estas fases de pesquisa citadas são importantes no conhecimento inicial das doenças, mas não em saúde pública. Já é de conhecimento notório que há sobreposição de territórios arteriais nas manifestações de riscos para eventos aterotrombóticos, portanto, o delineamento proposto é extremamente adequado (57). O escore de risco genético será avaliado quanto à sua associação, magnitude do efeito, precisão, portanto, com o real impacto clínico com a ocorrência desses eventos agudos. Análises de sensibilidade poderão ser efetuadas para explorar o comportamento dos escores genéticos em relação aos eventos ateroscleróticos agudos. Mais ainda, avaliar eventos ateroscleróticos agudos ditos ou denominados periféricos agrega valor substancial para a prevenção de eventos nos territórios arteriais aórticos e de vasos ditos periféricos, em extremidades, o que também adiciona impacto em qualidade de vida, por exemplo, em termos de amputações de membros potencialmente evitáveis. Por fim, um estudo pragmático deve ser delineado para englobar o amplo espectro dos eventos aterotrombóticos agudos, portanto, aumenta a robustez por garantir elevado poder estatístico, e nasce com princípio da generalização (validação externa).

7.4. Variáveis e Desfechos

Por ser estudo de caso-controle, não haverá seguimento para ocorrência de eventos clínicos. Desse modo, vamos avaliar as exposições a fatores de risco tradicionais em combinação com os dados genômicos (escore de risco poligênico), tanto nos casos quanto nos controles, e essas exposições serão expressas como razões de chances (OR). Modelos de regressão logística múltipla serão construídos para ajuste e determinação das forças de associação entre as variáveis demográficas, fatores de risco tradicionais e dados genéticos: Sexo, idade, etnia, peso, índice de massa corporal, tabagismo, diabetes, hipertensão arterial, obesidade, ansiedade e depressão, dieta inadequada, sedentarismo, consumo de bebida alcoólica, e relação apolipoproteína B/A1 (ApoB/ApoA1). Para cada variável de associação com maior chance de DCV (OR significante), será calculado um risco atribuível, para estimar a fração de risco atribuível ao componente genético (PRS) e às demais variáveis clínicas e demográficas.

7.5. Fonte de dados e mensuração

Os dados serão coletados em questionário eletrônico próprio (e-CRF), a ser desenvolvido e disponibilizado por meio do sistema REDCap. Neste formulário, serão inseridos todos os resultados de variáveis clínicas e demográficas (supracitados), além dos resultados de PRS e demais testes laboratoriais (HbA1c, apolipoproteína A1 sérica, apolipoproteína B sérica, colesterol sérico, colesterol total e frações (HDL, LDL e

• Rua Treze de Maio, 1.815 • Paulista
• CEP 01327 001 • São Paulo • SP
• Fone: 11 3549 1976 • Fax: 11 3549 0394

VLDL) séricos, triglicérides séricas, creatinina sérica, sódio sérico, potássio sérico, sódio na urina, potássio na urina, creatinina em amostra isolada de urina, albuminúria em amostra isolada de urina).

A avaliação genética será realizada por meio da associação de *Low-covering Whole Genome Sequencing* (cobertura 0,5-5x) e *Whole Exome Sequencing* (média de cobertura 30x). A partir deste teste será disponibilizado o PRS, conforme descrito a seguir.

7.5.1. Extração de dna das amostras

O DNA das amostras de sangue será extraído por processo automatizado (QIASymphony, utilizando o kit QIAmp DNA Mini Kit – Qiagen) seguindo as instruções dos fabricantes. O DNA extraído será analisado quanto sua quantificação pelo Qubit Fluorometer (Thermo Fisher) e será mantido – 20ºC até seu uso.

7.5.2. Construção das bibliotecas e sequenciamento do exoma e genoma low pass 1x

Para a construção das bibliotecas de exoma e genoma Low pass 1x será seguido o protocolo de preparo da empresa Twist Bioscience com fragmentação enzimática (Enzymatic Fragmentation and Combinatorial Dual Indices) com a utilização de 50 ng de input de DNA. A construção da biblioteca de exoma por sondas de captura híbrida levará em torno de 26 horas para sua finalização, e para o genoma Low pass 1x, levará em torno de 4 horas para a finalização.

Os DNA serão sequenciados em plataforma NovaSeq (Illumina) utilizando o kit NovaSeq S4 com 300 ciclos (2 x 150 bp - paired end). Os dados da corrida e controle de qualidade serão monitorados no programa NovaSeq control software.

Breve resumo do preparo do Exoma completo (WES):

(1)Fragmentação enzimática; (2) Reparo das extremidades; (3) Ligação dos adaptadores; (4) Purificação para retirar adaptadores não ligados; (5) Amplificação e indexação das amostras; (6) Avaliação da biblioteca por Qubit HS Sensitivity e TapeStation D1000; (7) Hibridização por sondas de WES; (8) Captura com beads de streptavidina; (9) Amplificação das regiões capturas; (10) Avaliação e quantificação da biblioteca final por Qubit HS Sensitivity e TapeStation D1000 High; (11) Normalização das bibliotecas finais para 2 nM.

Breve resumo do preparo do genoma Low pass 1x:

Fragmentação enzimática; (2) Reparo das extremidades; (3) Ligação dos adaptadores; (4) Purificação para retirar adaptadores não ligados; (5) Amplificação e indexação das amostras; (6) Avaliação da biblioteca por Qubit HS Sensitivity e TapeStation D1000; (7) Normalização das bibliotecas finais para 2 nM.

Os DNA serão sequenciados em plataforma NovaSeq (Illumina) utilizando o kit NovaSeq S4 com 300 ciclos (2 x 150 bp - paired end). Os dados da corrida e controle de qualidade serão monitorados no programa NovaSeq

• Rua Treze de Maio, 1.815 • Paulista
• CEP 01327 001 • São Paulo • SP
• Fone: 11 3549 1976 • Fax: 11 3549 0394

control software. A leitura do sequenciamento será paired end com 300 pares de bases (150 pares de bases forward e 150 pares de bases reverse).

As métricas de sequenciamento devem apresentar cluster passing filter >70%, flow cell occupation >70%, Q30 score > 75%, Intensidade das bases > 1000, Error Rate abaixo de 3%, Phas/Prephas abaixo de 0,5%.

A validação do protocolo e pipeline de análise do sequenciamento de genoma completo de *low covering* será realizada seguindo critérios rigorosos de qualidade. Serão utilizadas 20 amostras, que serão avaliadas internamente pelo Grupo Fleury e por laboratório externo de referência. O arquivo de chamada de variantes (arquivos vcf) do sequenciamento interno e do sequenciamento realizado pelo laboratório de referência serão comparados para cada amostra, permitindo definir os parâmetros de especificidade e sensibilidade, bem como a identificação de variantes falso positivas e falso negativas. O sequenciamento de exoma completo é uma técnica validada e rotineiramente realizada no Grupo Fleury.

7.5.3. Análise dos dados gerados

Os dados gerados serão processados em dois pipelines distintos. O pipeline do sequenciamento completo d Exoma já está implantado e funcional. O pipeline que irá processar os dados de genoma completo (WGS) está em fase de desenvolvimento. Ambos os pipelines são de responsabilidade do Grupo de Ciência de Dados e Bioinformática do P&D (Grupo Fleury).

Os pipelines estão baseados no sistema Dragen v.3.6.4 da Illumina instalado em servidores localizados na sede do Grupo Fleury. Ao término das corridas, o pipeline irá realizar a etapa de demultiplexação, criação dos arquivos FASTQ (contendo os reads) para cada amostra, alinhamento dos reads ao genoma humano referência (Hg19), criação dos arquivos BAM contendo os reads alinhados e criação dos arquivos VCFs contendo as variantes identificadas (SNPs, INDELS, CNV). Após a finalização dessa etapa, os arquivos BAMs e VCFs gerados serão armazenados na nuvem (AWS) e em seguida enviados para a plataforma da empresa Emedgene para a anotação/classificação das variantes encontradas nos genes: *LDLR, APOB, PCSK9, LDLRAP1, ABCG5, ABCG8, APOE, LIPA*. Os dados gerados nas variantes monogênicas associadas a dislipidemias familiares serão tratados, analisados e laudados por profissionais especializados (bioinformatas, analistas e geneticistas). Os dados do genoma Low pass 1x serão submetidos ao método de imputação, que garante a qualidade na identificação de variantes e serão utilizados em conjunto com a chamada de variantes do exoma pela equipe de sistemas e dados para a calibração do PRS. Atualmente, existe grande evidência, inclusive comparativa, sobre o uso de técnicas de imputação e o uso apenas do WGS completo(58–61). Trata-se de técnica com alta sensibilidade e boa acurácia, extensivamente empregada para lidar com falta de tempo e recursos para a realização de WGS completo. Um estudo mostrou que, na maioria dos casos, a variabilidade do PRS devido à imputação é pequena (mudança de classificação <5 percentil) e não influencia a interpretação da pontuação(62). Mesmo assim,

• Rua Treze de Maio, 1.815 • Paulista
• CEP 01327 001 • São Paulo • SP
• Fone: 11 3549 1976 • Fax: 11 3549 0394

sugere alguns pontos de cautela, como a necessidade de se aplicar processos de imputação determinísticos ou a média de múltiplas iterações de processos de imputação estocásticos para gerar resultados de PRS(62). Ademais, existe um risco com a diversidade da população estudada é pequena ou quando se utiliza bancos de imputação de populações outras que não aquela da região do estudo(63). Adicionalmente, o PRS demonstrou melhorar a estratificação de risco para DCV aterosclerótica e identificar pacientes que podem obter maior benefício da prevenção primária e secundária. O risco capturado pelo PRS parece amplamente independente dos fatores de risco tradicionais. Entretanto, o caminho para implementação do PRS como ferramenta clínica ainda exige alguns cuidados, como: melhorar a capacidade de generalização do PRS incluído estudos de avaliação WGAS em outras populações que não a europeia, já extensivamente estuda; padronizar o método para DCV para que este possa ser mais facilmente inserido em guidelines da área; consolidar a comunicação de PRS associado a medidas de mudança comportamental para melhor benefício clínico(64). Estudos recentes que utilizaram WGS low-pass para imputação de dados e geração de PRS mostraram uma boa acurácia da técnica em grandes bancos de dados de populações miscigenadas, para DCV e alguns canceres(65,66). Também, existe evidência de custo-efetividade do procedimento com WGS low-pass, com maior otimização analítica frente aos arrays(67). Estamos atentos a essas recomendações e ressaltamos que as melhores técnicas analíticas serão utilizadas. Além disso, esperamos poder contribuir para a generalização do uso e aplicação do PRS, uma vez que iremos agregar dados de uma população representativa e miscigenada como é a brasileira.

7.5.4. Calibração e cálculo de PRS para doenças cardiovasculares

A literatura do cálculo de score poligênico (PRS) é relativamente recente. Apesar disso, sua evolução cresce em ritmo acelerado, principalmente devido ao aumento de datasets de dados genéticos que permitem a proposição de novos métodos de cálculo e, ao crescimento de sua utilização para predição de doenças, fruto do aumento de sua precisão em pesquisas baseadas em casos controle e população em geral (68).

O PRS agrega os efeitos de variantes genéticas em um único número que prevê a predisposição genética para um fenótipo. Os PRS são tipicamente compostos de centenas a milhões de variantes genéticas (geralmente SNPs). Para cada indivíduo, o número de alelos de risco computados em cada variante é somado e pesado pelo valor estimado dos efeitos obtidos (log odds ratio para características com valores binários ou coeficientes Betas para características com valor contínuo (68)) obtidos de estudos genômicos em larga escala (GWAS), como pode ser visto na equação 1:

$PRS= β_i k_i+ β_{(i+1)} k_{(i+1)} +⋯ +β_n k_n$ 1)

sendo que $β_i,β_{(i+1)},…,β_n$ são os efeitos obtidos, $k_i,k_{(i+1)},…,k_n$ a quantidade de alelos de risco computados em cada variante (0, 1 ou 2) e $i,i+1,…,n$ são os índices dos SNPs (69).

• Rua Treze de Maio, 1.815 • Paulista
• CEP 01327 001 • São Paulo • SP
• Fone: 11 3549 1976 • Fax: 11 3549 0394

7.5.5. Seleção e avaliação de modelos PRS

Para o cálculo e calibração do PRS para doenças cardiovasculares, modelos PRS prévios serão selecionados no PRS Catalog (70). O PRS Catalog é um banco de dados aberto de pontuações score poligênico PRS publicadas. Cada PRS é consistentemente anotado com metadados relevantes incluindo arquivos de pontuação, os quais contêm informação das variantes, alelos de risco (effect allele) e tamanho de efeito, além de anotações de como o PRS foi desenvolvido (método usado) e aplicado e avaliações de seu desempenho preditivo. Também será consultado o GWAS Catalog, uma base de dados pública com uma coleção curada de estudos GWAS (71). Este catálogo nos auxiliará na compreensão dos componentes genéticos relacionados as doenças avaliadas, identificando os principais SNPs.

Atualmente estão depositados no banco 22 PRS para doenças arteriais coronarianas (DAC), um para IAM e cinco para AVC isquêmico. Avaliaremos o desempenho de diferentes modelos do banco (com h2SNP >0.05) que foram desenvolvidos e testado em indivíduos com diferentes ancestralidades, principalmente a europeia, com objetivo de calibrá-los para a população brasileira, cuja miscigenação representa um desafio para a generalização do PRS na nossa população.

De forma paralela, será realizada a avaliação da ancestralidade por meio de Marcadores Indicativos de Ancestralidade (AIMSs). Para cada indivíduo será estimada a % da contribuição do componente ancestral do 1000G (AFR: Africana, AMR: Nativo Americano, EAS: Leste Asiático, EUR: Europeu e SAS: Sul Asiático) usando o software ADMIXTURE (72). Isto com a finalidade de avaliar a relação entre ancestralidade e o viés potencial em PRS desenvolvidos em populações com ancestralidade europeia, devido a diferenças populacionais no desequilíbrio de ligação e frequência alélica, pela deriva genética.

Serão utilizados 192 AIMs do tipo SNVs previamente validado por Santos et al (2016)(73), para a determinação da ancestralidade genética de populações brasileiras. Estes marcadores foram validados para identificar e quantificar com precisão a ascendência de indivíduos latino-americanos ou hispanos/latinos dos USA. Como painel de referência para os estudos de ancestralidade serão considerados indivíduos do Projeto de Diversidade do Genoma Humano (HGDP)- Pima, Maya como ameríndios, e do projeto HapMap- Africanos YRI (Yoruba em Ibadan, Nigéria), LWK (Luhya em Webuye, Quênia), ASW (Americanos de ascendência africana em SW, USA); Europeu CEU (Residentes de UTAH-CEPH- com ascendência da Europa do Norte e Ocidental) e TSI (Toscana na Itália).

A análise de ancestralidade através do programa ADMIXTURE, será realizada usando os parâmetros relatados por Santos et al (2016)(73), utilizando 200 réplicas bootstrap (padrão) e k=3 (número de populações assumidas para a análise). Com a finalidade de testar o nosso pipeline de análise, serão reproduzidos os resultados obtidos por Santos et al (2016)(73) utilizando os dados genéticos do projeto EpiGen-Brasil depositados no European

• Rua Treze de Maio, 1.815 • Paulista
• CEP 01327 001 • São Paulo • SP
• Fone: 11 3549 1976 • Fax: 11 3549 0394

Genome-phenome Archive (EGA, https://www.ebi.ac.uk/ega/), hospedado pelo EBI, sob o número de acesso EGAS00001001245.

7.5.6. Seleção dos SNPs para cálculo de PRS

Após a imputação do WGS-LP (1x), serão consideradas amostras com taxa de genotipagem >0.99 e as variantes SNPs que preencham os 3 critérios a seguir serão mantidas para o cálculo de PRS: 1) boa qualidade de imputação (INFO>0.8); 2) frequência do alelo menor (MAF) >1% e 3) p-valor maior do que 1x106 em testes de equilíbrio de Hardy-Weinberg específicos de ancestralidade. SNPs ambíguos, mismatched, duplicados e localizados em cromossomos sexuais serão descartados (74). Nos estudos de genética de populações é comum testar se as frequências genotípicas observadas estão de acordo com as esperadas nas hipóteses do Equilibrio de Hardy-Weiberg, para tal será empregado os testes do software PLINK descritos por Chang C.C.(75).

Para esta avaliação de controle de qualidade de dados, será usada a ferramenta PLINK (76). Esta ferramenta, implementada em C/C++, permite a manipulação e análise de dados de GWAS de maneira facilitada e otimizada, atuando em diversas etapas, entre elas: controle de qualidade de dados, gestão dos dados, estatísticas de resumo, estratificação de populações, análises de associação e estimação de IBD (identity-by-descent).

7.5.7. Cálculo e calibração de PRS

Para o cálculo de PRS, o método mais comumente utilizado é o Clumping and Threshold (C+T), também conhecido como Pruning e Threshold (P+T) (77). O Clumping seleciona as variáveis mais significantes iterativamente, calcula as correlações entre as variantes próximas dentro de uma região genética (w_c) e remove todas as variantes dentro desta região com um valor de correlação acima de um limite definido pela variável r_c^2. O Threshold por sua vez, consiste em remover as variantes que contêm p-valores (obtidos do teste de hipótese das regressões lineares entre o número de alelos de efeito de cada amostra e a característica de interesse (69,78)) que ultrapassam o nível de confiança escolhido (p > p_T). Para cada amostra, o PRS é então calculado como a soma dos efeitos encontrados para cada alelo de risco em cada SNP. Este método é implementado em sua forma padrão pela ferramenta PRSice-2 (74).

Os métodos utilizados para calcular o PRS buscam encontrar um compromisso estatístico entre sinal e ruído. Neste contexto, como os valores dos parâmetros (w_c, r_c^2, p_T) do método C+T são arbitrários, o método apresenta dificuldades em encontrar os valores ótimos dos parâmetros para maximizar a qualidade das predições. Desta forma, outros métodos foram propostos para sanar as dificuldades apresentadas, como:

• Rua Treze de Maio, 1.815 • Paulista
• CEP 01327 001 • São Paulo • SP
• Fone: 11 3549 1976 • Fax: 11 3549 0394

métodos empregando regressão LASSO (78), estatística bayesiana (79) e machine learning, como o SCT (77,80). Alguns destes métodos podem ser vistos mais detalhadamente a seguir:

lassosum: método utilizado para suplementar o cálculo de PRS com informações externas de desequilíbrio de ligação (78). Pode ser utilizado em R ou diretamente na linha de comando para sistemas UNIX. Nesta metodologia, baseada em regressão penalizada (LASSO), busca-se estimar os tamanhos dos efeitos dos SNPs (os coeficientes da regressão) ao minimizar a função de perda com um penalizador (neste caso, 2λ∑_i▒〖|β_i |〗, ou a norma L1 dos coeficientes β da regressão), geralmente estimado utilizando validação cruzada.

LDPred2: método que estima tamanhos de efeitos causais médios posteriores a partir de estatísticas-resumo de GWAS (79). Posteriormente, o método filtra as variantes baseado nas suas correlações e similaridades associadas às outras variantes da população de referência (81). Assim, assume-se uma probabilidade a priori para a arquitetura genética e a informação de desequilíbrio de ligação, possibilitando a análise de características de interesse e doenças com uma ampla gama de estruturas gênicas. A metodologia do pacote está implementada no pacote bigsnpr para R (80).

Stacking C+T (SCT): o método utiliza uma regressão penalizada para encontrar uma combinação linear dos múltiplos valores dos parâmetros C+T (77). Assim como o método de estatística bayesiana, o SCT também está implementado no pacote bigsnpr para R.

MultiPRS: um método desenvolvido para análise de PRS em populações miscigenadas (82).

O advento da biologia computacional trouxe inúmeros desafios relacionados à análise de grandes quantidades de dados biológicos, armazenamento e processamento. No âmbito de GWAS, diversas ferramentas para facilitar as análises de SNPs relacionados a características de interesse já foram implementadas. O PLINK, uma das ferramentas mais utilizadas, calcula o PRS utilizando a equação (1) com um fator de ajuste no denominador a fim de ponderar os SNPs potencialmente faltantes (68). Este cálculo é feito conforme mostra a equação (2), em que S_i é o tamanho do efeito do SNP i; G_ij é o número de alelos "efeito" observados na amostra j; P é a ploidia da amostra (2 para humanos); N é o número total de SNPs inclusos no PRS e M_j é o número de SNPs não-faltantes na amostra j. Para amostras com genótipos faltantes para o SNP i, o MAF populacional multiplicado pela ploidia (〖MAF〗_i*P) é utilizado no lugar de G_ij.

PRS〗_j=iNS_i*GijP*Mj (2)

No presente trabalho, inicialmente será feito um estudo de GWAS (Genome-wide association studies) para a população brasileira a fim de obter os efeitos dos SNPs e variáveis externas (sexo, possivelmente ancestralidade, idade) para cada uma das condições clínicas. Em seguida, utilizaremos os métodos de C+T, Regressão LASSO, Estatística Bayesiana e SCT para encontrar os melhores parâmetros de corte na seleção dos

• Rua Treze de Maio, 1.815 • Paulista
• CEP 01327 001 • São Paulo • SP
• Fone: 11 3549 1976 • Fax: 11 3549 0394

SNPs e realizar o cálculo de PRS para a população brasileira. A métrica utilizada para comparação entre os métodos será feita pela AUC (area under curve) da curva ROC resultante, sensibilidade e especificidade.

Por fim, para minimizar o efeito da quantidade reduzida da amostra da população brasileira no cálculo do PRS, utilizaremos um PRS multiétnico por meio da combinação linear entre PRS população brasileira e um PRS população europeia a ser definido, como descrito na fórmula abaixo (82):

$$PRS = \alpha_1 PRS_{BR} + \alpha_2 PRS_{EU}$$

em que α_1 e α_2 são os pesos dos PRS da população brasileira e população europeia, respectivamente.

Conforme descrito em Márquez-Luna et al 2017, o método proposto é um modelo mistura. Desta forma, os pesos (alpha) apresentados serão estimados a partir do conjunto de validação da análise, através de validação cruzada de 10 "folds". Em cada "fold" o peso de cada população no R^2 do PRS será estimado, e ao final a média dos pesos em cada fold será utilizada como o peso final.

Os demais exames bioquímicos serão realizados conforme técnica padronizada pelos laboratórios da rede conveniada ao laboratório Fleury (**Quadro 2**).

• Rua Treze de Maio, 1.815 • Paulista
• CEP 01327 001 • São Paulo • SP
• Fone: 11 3549 1976 • Fax: 11 3549 0394

Quadro 1: Exames bioquímicos, estabilidade e metodologia

Descrição do exame	SIGLA	Estabilidade Refrigerada	Estabilidade Congelada	Metodologia
Hemoglobina glicada, sangue total	HGBGLIC	(2-8 ºC): 7 dias;	(-20 ºC): 30 dias;	Cromatografia em Coluna de troca iônica em sistema de HPLC
Apolipoproteína A-1, soro	APOLIPA	(2-8 ºC): 8 dias;	(-20 ºC): 2 meses	Imunoturbidimétrico
Apolipoproteína B, soro	APOLIPOB	(2-8 ºC): 8 dias;	(-20 ºC): 2 meses	Imunoturbidimétrico.
Colesterol, soro	COLEST	(2-8 ºC): 7 dias;	(-20 ºC): 3 meses.	Enzimático colorimétrico
HDL Colesterol, soro	HDLCOL	(2-8 ºC): 7 dias;	(-20 ºC): 3 meses.	Enzimático colorimétrico.
Colesterol, Fração LDL, soro	LDLTE	(2-8 ºC): 7 dias;	(-20 ºC): 3 meses.	Cálculo baseado nas fórmulas de Friedewald e Martin
Colesterol, fração VLDL, soro	VLDLTE	(2-8 ºC): 7 dias;	(-20 ºC): 3 meses.	Fórmula de Martin e colaboradores
Triglicérides, soro	TRIG	(2-8 ºC): 7 dias;	(-20 ºC): 1 ano.	Enzimático colorimétrico
Creatinina, urina	CREATUR	(2-8 ºC): 5 dias;	(-20 ºC): 1 ano.	Cinético colorimétrico
Sódio, soro	NA	(2-8 ºC): 7 dias	(-20 ºC): 6 meses.	Potenciométrico
Potássio, soro	K	(2-8 ºC): 7 dias;	(-20 ºC): 6 meses	Potenciométrico
SÓDIO, AMOSTRA ISOLADA, URINA	NAUR	(2-8 ºC): 7 dias;	(-20 ºC): 6 meses	Potenciométrico
Potássio, urina	KUR	(2-8ºC): 7 dias;	(-20 ºC): 3 meses.	Potenciométrico

• Rua Treze de Maio, 1.815 • Paulista
• CEP 01327 001 • São Paulo • SP
• Fone: 11 3549 1976 • Fax: 11 3549 0394

	Creatinina, amostra isolada, urina	CREATISO	(2-8 ºC): 5 dias;	(-20 ºC): 1 ano.	Cinético colorimétrico
	Albuminúria, amostra isolada, urina	MCRALBISO	(2-8 ºC): 14 dias;	(-20 ºC): 6 meses;	Ensaio imunoturbidimétrico

• Rua Treze de Maio, 1.815 • Paulista
• CEP 01327 001 • São Paulo • SP
• Fone: 11 3549 1976 • Fax: 11 3549 0394

Tanto para os testes genéticos, quanto para os demais testes bioquímicos, será enviado kit completo contendo tubos, agulhas, *stop blood*, garrote, e material de suporte, para cada paciente incluído, com as respectivas etiquetas de identificação. Todas as análises serão realizadas em São Paulo, nas unidades de análise do Laboratório Fleury. Para tanto, as amostras serão estocadas nos centros participantes em freezers a -20ºC, para posterior remessa controlada para São Paulo. Espera-se a quantidade de uma remessa mensal com as amostras coletadas por cada centro, para manter um princípio de economicidade e sustentabilidade do projeto.

A análise de variantes e o resultado de testes genéticos serão realizados em parceria entre laboratório Fleury S.A e HAOC, salientando que o pesquisador principal de cada centro participante e/ou pesquisador assistente será responsável por informar os resultados aos pacientes sempre que solicitado. O acordo de parceria entre HAOC e Fleury é firmado por termo de cooperação, o qual será anexado ao plano de trabalho completo. Cabe ressaltar que a base de dados gerada será compartilhada com o Ministério da Saúde para compor a base de dados genômicos do Programa Nacional de Genômica e Saúde de Precisão - Genomas Brasil. Além disso, todas as amostras advindas de teste genético bem como o grande volume de dados gerados a partir delas serão armazenados durante todo o período do estudo e fornecidas ao Ministério da Saúde, sempre que solicitadas. É importante ressaltar que o acesso aos dados clínicos e demográficos tabulados, bem como acesso às amostras de DNA, plasma e/ou soro fica garantido aos envolvidos neste projeto, a saber: Hospital Alemão Oswaldo Cruz, Ministério da Saúde e Laboratório Fleury S.A., inclusive por período superior ao de duração do estudo. Esse acesso se faz necessário para o ajuste de análises, proposição de análises exploratórias, análises de subgrupos e para o eventual desenvolvimento de testes ou produtos com finalidade diagnóstica, seguindo as minutas para propriedade intelectual do Programa Nacional de Genômica e Saúde de Precisão - Genomas Brasil. Ressalta-se que o presente projeto e as parcerias nele estabelecidas estarão de acordo com as políticas e diretrizes do Programa Nacional de Genômica e Saúde de Precisão - Genomas Brasil.
Como o escopo do projeto está focado na construção de um *poligenic risk score* para variantes associadas à DCV, tais informações de cunho genético serão divulgadas aos pacientes apenas após a finalização e publicação do estudo, quando o risco atribuível exclusivamente a dados genéticos na população brasileira será conhecido e ajustado por variáveis demográficas e fenotípicas. Conforme Manual de Pendências da CONEP, página 39:

• Rua Treze de Maio, 1.815 • Paulista
• CEP 01327 001 • São Paulo • SP
• Fone: 11 3549 1976 • Fax: 11 3549 0394

“O resultado de qualquer exame, não somente os de natureza genética, deve ser assegurado ao participante de pesquisa sempre que solicitado por ele, salvo quando essa informação interferir no desfecho da pesquisa”. Nesse sentido, por se tratar de condição de saúde de influência poligênica, o compartilhamento apenas dos testes genéticos é pouco conclusivo, sem o ajuste pelas variáveis clínicas, e pode gerar alarde, recusas e desistências quanto à participação na pesquisa. Da mesma forma, em relação a achados secundários ou incidentalomas nos testes genéticos, o Parecer Técnico da Sociedade Brasileira de Genética Médica e Genômica sobre testes genéticos (3) recomenda que “*informações sobre: achados secundários, variantes em heterozigose para doença recessiva (status de portador), farmacogenômica, entre outros*” devam constar no laudo do exame apenas se o paciente tenha requerido e consentido em Termo de Consentimento Livre e Esclarecido ser informado sobre tais condições. Mesmo assim, sempre que solicitado, os testes serão compartilhados, conforme legislação ética aplicável, porém não será prática do projeto a divulgação espontânea de achados genéticos sem o devido ajuste por variáveis clínicas.

Após a finalização do estudo e publicação dos resultados, os pacientes serão comunicados diretamente pela equipe de pesquisadores do projeto dos exames genéticos de acordo com resultados identificados: 1) para os casos onde variantes monogênicas forem detectadas, forneceremos laudo com descrição da mesma e realizaremos orientações para triagem de outros familiares possivelmente acometidos pela mesma variante; para todos os outros pacientes (inclusive aqueles nos quais forem detectadas variantes monogênicas), será informado o impacto do resultado do *Polygenic Risk Score* com devidas correções para os demais fatores de risco cardiovascular fenotípicos, informando ao paciente o peso específico do componente genético como contribuinte para a doença cardiovascular.

Avaliaremos variantes monogênicas associadas a dislipidemias hereditárias, ou seja, condições diretamente relacionadas às DCV (que são as condições-alvo deste projeto). A hipercolesterolemia familiar representa a principal causa monogênica de doença arterial coronária aterosclerótica(83). A prevalência da hipercolesterolemia familiar é de aproximadamente 1:313 na população geral, sendo 10 vezes mais frequente em indivíduos com doença cardíaca isquêmica(84). No estudo de Khera et al, uma mutação para hipercolesterolemia familiar estava presente em 1,7% de 2.081 pacientes com infarto agudo do miocárdio precoce, aumentando o risco desse desfecho em 3,8 vezes (95%CI 2.1 – 6.8; $p < 0.001$)(85). Os genes implicados na hipercolesterolemia familiar são em ordem decrescente de frequência o *LDLR*, *APOB* e *PCSK9*. Mutações raras no *LDLRAP1* também podem causar uma forma autossômica recessiva dessa desordem. Finalmente, mutações nos genes *ABCG5, ABCG8, APOE* e *LIPA*, também incluídos na nossa seleção, podem resultar em fenótipos semelhantes ao fenótipo de hipercolesterolemia familiar (fenocópias)(86). Portanto, a seleção dos genes acima decorreu da importância da hipercolesterolemia familiar como grande causa monogênica de DCV aterosclerótica, sobretudo coronária. Outras causas monogênicas de doença arterial coronária prematura

• Rua Treze de Maio, 1.815 • Paulista
• CEP 01327 001 • São Paulo • SP
• Fone: 11 3549 1976 • Fax: 11 3549 0394

incluem por exemplo mutações nos genes *APOA1* e *ABCA1* (respectivamente responsáveis pela deficiência de *APOA1* e doença de Tangier associadas a níveis extremamente baixos de HDL-C), mas são muito raras(87).
A análise de achados acidentais, como sugerido pelo ACMG, demanda análise detalhada por equipe treinada e tempo. Dessa forma, pela estrutura necessária, recursos físicos e humanos e, também, pelo prazo restante para a execução deste projeto, não seria factível a realização de tal análise. Sendo assim, nossa proposta é avaliar, nesse momento, os achados monogênicos importantes relacionados à hipercolesterolemia e, numa eventual continuidade do projeto, propor ações para lidar com outros achados incidentais.

7.5.8. Controle de viés

Os critérios de elegibilidade para casos e controles serão rigorosamente seguidos para reduzir viés de seleção. Os critérios definidos para primeiro evento de DCV serão padronizados e todos os centros serão treinados para a correta identificação, registro de dados e relato de informações. Apesar de não ser um estudo pareado, a relação entre as variáveis será ajustada por modelos logísticos multivariados. A adoção do desenho não pareado se deve: 1) o ajuste por múltiplas variáveis por meio de regressão logística acoplada às devidas análises por subgrupo é método aceito e muitíssimo elegante e robusto para o tipo de análise proposto; 2) A seleção de controles no contexto do nosso cronograma pode ser um impeditivo importante ao bom andamento do projeto e à velocidade com que as amostras biológicas serão enviadas e analisadas (temporalidade do triênio PROADI-SUS); e 3) Sabemos que o pareamento em si, em um delineamento caso- controle, não elimina fatores de confusão (88). Adicionalmente, a escolha de variáveis de pareamento que nãosão realmente fatores de confusão reduz a eficiência deste tipo de modelagem.
Desta forma, no modo não-pareado proposto, análises de regressão logística binária múltipla não condicional poderiam ser aplicadas controlando (ajustando-se) por covariáveis potenciais fatores de confusão advindos da literatura e por covariáveis que poderiam ter definido o pareamento, caso o modelo pareado tivesse sido escolhido.
Quanto aos testes genéticos e clínicos, estes serão realizados pela mesma rede de laboratórios, garantindo a padronização dos métodos.

7.5.9. Tamanho amostral

Diante de um modelo de doença complexa ou poligênica a ser estudado, i.e., a presença de variantes em heterozigose somadas entre si para representar potencial patogênico, os cálculos estatísticos de tamanho amostral assumem um padrão de herança dominante, com delineamento de estudo caso-controle não-pareado 1:1, uma amostra de 1867 casos e de 1867 controles teria poder estatístico de 90% para detectar OR

• Rua Treze de Maio, 1.815 • Paulista
• CEP 01327 001 • São Paulo • SP
• Fone: 11 3549 1976 • Fax: 11 3549 0394

genético de 1.3, assumindo frequência alélica em 10% neste caso-controle não-pareado 1:1 de fator genético. Considerou-se prevalência de 10% da doença (IAM/AVC/DAP) na população alvo, nível de significância alfa de 5%, teste de hipótese bilateral. Cálculo de tamanho amostral foi efetuado via QUANTO versão 1.2.4 (4,5).

O racional de se usar o padrão de herança autossômico dominante para cálculo do tamanho amostral reside no fato de que não precisamos de variantes em homozigose ou heterozigose compostas no mesmo gene para contribuir para o risco aumentado da doença (que seria o padrão autossômico recessivo). Na presença de um SNP, mesmo que em heterozigose, tal variante tem poder para contribuir para o aumento do risco per se. Não é do nosso conhecimento que existem outras inciativas publicadas na literatura para as DCV ateroscleróticas com tamanhos amostrais exorbitantes. Dessa forma, para assumirmos um pragmatismo no cálculo amostral, adotamos como premissa um padrão hereditário do tipo autossômico dominante e uma diferença de 0,3 no OR. Para lidar com o fato de ter adotado uma premissa de herança dominante, foi utilizado um poder estatístico de 90%, para sermos mais conservadores e garantirmos o maior tamanho amostral possível para o cenário proposto. Com um tamanho amostral de, aproximadamente, 4000 indivíduos, teremos condições para avaliar as possíveis influências poligênicas na patogênese das DCV ateroscleróticas. Outro estudo na área da cardiologia utilizou, para a América Latina, 584 casos e 665 controles, para avaliar, em análise ajustada, a relação entre escore de risco genético e infarto do miocárdio(89).

7.5.10. Variáveis quantitativas e análise estatística

O teste de equilíbrio genético de Hardy-Weinberg será avaliado no grupo controle via teste qui-quadrado ou teste exato de Fisher (90). Para estimar a associação entre os polimorfismos genéticos e risco de doença cardiovascular, análises de regressão logística não-condicional univariada e múltipla serão conduzidas (91–93). Inicialmente, análises de regressão logística binária univariada serão conduzidas. Em seguida, covariáveis que apresentarem valor-p < 0.20 nas análises de regressão univariada serão consideradas em análise de regressão logística múltipla com seleção de variáveis segundo técnica de eliminação *backward* (92,93). Valor-p < 0.05 será utilizado como critério de retenção de variáveis no modelo.

Na seleção *backward* será utilizada uma variante da eliminação *backward* que considera o máximo possível de tamanho da amostra à medida que o número de covariáveis no modelo diminui a cada etapa deste processo iterativo (94). Eventualmente covariáveis julgadas como fatores de confusão pelo pesquisador poderão ser forçadas no modelo final de regressão via *backward*.

Análises adicionais de regressão logística múltipla poderão ser também conduzidas utilizando-se uma variante do algoritmo *Purposeful Selection* descrito por Bursac *et al* (95,96).

A suposição de linearidade na escala logit (log-odds) entre cada covariável quantitativa e a variável resposta binária em análise de regressão logística binária será avaliada com a construção de "*Smoothed Scatter Plots*"

• Rua Treze de Maio, 1.815 • Paulista
• CEP 01327 001 • São Paulo • SP
• Fone: 11 3549 1976 • Fax: 11 3549 0394

e método dos polinômios fracionais (92,97). Quando a suposição não for satisfeita, covariáveis quantitativas serão categorizadas para utilização em regressão logística utilizando-se pontos de corte segundo a literatura, tercis da distribuição ou cut off ótimo obtido de curva ROC (*Receiver Operating Characteristic Curve*), o que for julgado mais apropriado. No caso de ponto de corte ótimo, o mesmo será definido como aquele que maximiza o índice de Youden (97,98).

A presença de multicolinearidade será avaliada via estimação de fatores de inflação de variância ("*Variance Inflation Fator*" (VIF)). Valores de VIF > 2.5 servirão como indicadores de considerável multicolinearidade em análise de regressão logística (92). *Odds ratios* (OR) brutos e ajustados com IC95% serão reportados.

Possíveis interações entre covariáveis que permanecerem no modelo final de regressão serão avaliadas, incluindo termos de interação no modelo (91,92,99).

A calibração e a habilidade discriminatória do modelo final de regressão logística múltipla serão avaliadas via teste de Hosmer-Lemeshow e a área sob a curva ROC(AROC) respectivamente. Valor-p > 0.05 para o teste de Hosmer-Lemeshow indica que modelo estaria calibrado, ou seja, as probabilidades preditas pelo modelo refletem adequadamente a ocorrência do evento nos dados. Como regra geral, será considerado a seguinte interpretação para a AROC: AROC=0.5:discriminação ausente, 0.5<=AROC<0.7 discriminação de pequena relevância, 0.7<=AROC<0.8: discriminação aceitável, 0.8<=AROC<0.9 discriminação excelente; AROC>=0.9: discriminação quase perfeita (93).

Risco Atribuível à População será estimado.

Na caracterização da população do estudo, variáveis categóricas serão descritas com contagens e proporções e comparadas com teste qui-quadrado de Pearson ou com o teste exato de Fisher (100). As variáveis quantitativas de distribuição normal e assimétrica serão descritas como média (desvio padrão) ou mediana (intervalo interquartil) respectivamente (101). A normalidade será avaliada por inspeção visual de histogramas e aplicação de testes de normalidade se apropriado (102,103). Comparação destas variáveis do tipo contínuas serão efetuadas com teste t de student para amostras independentes ou alternativa não paramétrica, teste de Mann-Whitney (100).

Todas as análises estatísticas seguirão o princípio *complete case analysis*. Todos os testes de hipóteses serão bilaterais e valor-p<0.05 considerado estatisticamente significante. Análise estatística de dados será conduzida com SAS 9.4 (SAS Institute, Cary, NC).

8. QUESTÕES ÉTICAS

O presente estudo irá seguir todas as recomendações de boas práticas e ética em pesquisa, conforme estabelecido na Resolução CNS 466 de 12 de dezembro de 2012. Todos os participantes deverão assinar termo de consentimento livre e esclarecido (TCLE) de modo voluntário, respeitando todos os direitos dos

• Rua Treze de Maio, 1.815 • Paulista
• CEP 01327 001 • São Paulo • SP
• Fone: 11 3549 1976 • Fax: 11 3549 0394

participantes de pesquisa, incluindo o sigilo e confidencialidade. A todos os pacientes será explicada a pesquisa e, a qualquer momento, será assegurado o direito de retirada de consentimento.

Considerando que as DCV poligênicas podem ter pouca explicação relacionada à questão genética, divulgar os dados previamente, sem os devidos ajustes por variáveis fenotípicas, clínicas e demográficas pode ser precipitado, podendo gerar alarde, desistências e até falta de credibilidade na presente pesquisa. Sendo assim, os dados de risco atribuível à genética serão divulgados ao final da pesquisa, quando este estiverem devidamente ajustados (ver seção fonte de dados e mensuração acima).

O projeto será submetido ao CEP do HAOC como coordenador ético.

O fluxograma exibindo a metodologia e logística do estudo é descrito na **Figura 3** a seguir

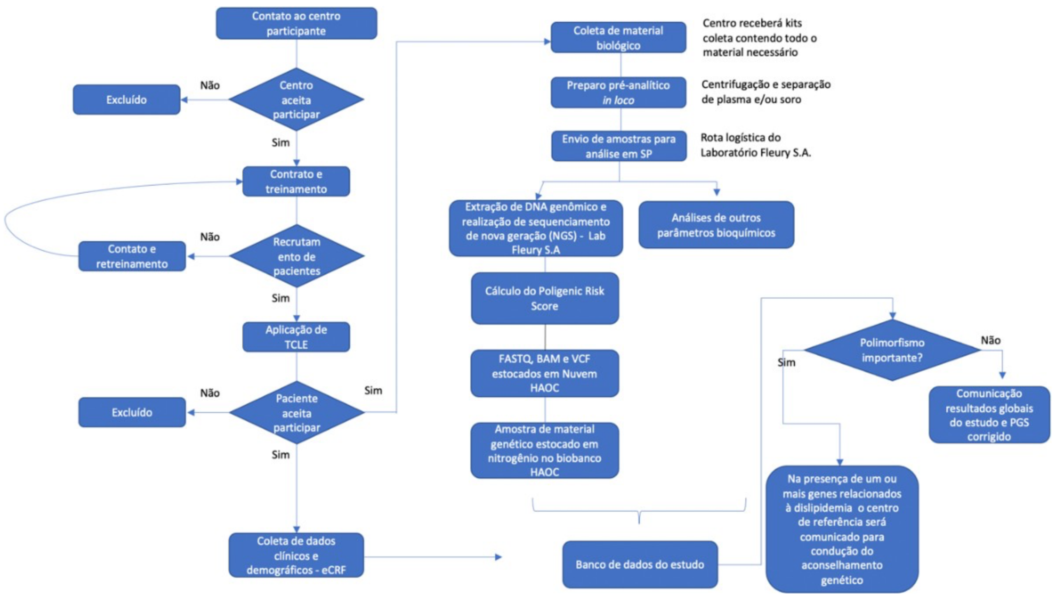

Figura 3: Fluxograma metodológico e logístico do estudo CV-GENES.

9. DIVULGAÇÃO DOS RESULTADOS E MONITORAMENTO

Serão realizados dois *workshops* virtuais para a promoção de capacitação, apresentação de convidados referência na área de DCV e genética, *brainstormings* sobre a pesquisa, levantamento de principais dificuldades e, também, aspectos positivos e divulgação de dados da pesquisa para Ministério da Saúde, especialistas profissionais de saúde convidados, centros participantes e pacientes. A proposta compreende até dois eventos, um preliminar e outro final, com capacidade para até 1000 (mil) participantes cada (considerando capacidade de suporte da plataforma de webinar e alcance em público), na modalidade virtual.

Em 15 de dezembro de 2022 (previsão), será realizado workshop com a participação de palestrantes do Brasil e de fora do país, de forma a propiciar um cenário crítico de construção do raciocínio científico por trás do

projeto CV-genes. A ideia é propiciar capacitações para o bom andamento do estudo, atrelando as experiências preliminares ao final do primeiro ano da pesquisa às experiências vividas por pesquisadores e entidades relevantes na área, como: Sociedade Brasileira de Genética Médica (SBGM), Projeto Genomas Brasil, Outros HEs do PROADI-SUS envolvidos com o Genomas Brasil, Universidade de Mc Master, Genomics England, entre outros.

Em 30 de novembro de 2023 (previsão), serão divulgados os dados do estudo a profissionais da saúde, entidades importantes da área e participantes do estudo. A intenção é propiciar um cenário diferente do workshop preliminar, no qual o foco sejam os dados obtidos pelo estudo, com menção a dificuldades, pontos de melhoria e aspectos positivos. Nesse evento pretende-se dar ênfase aos centros participantes com melhor adesão ao protocolo, com agradecimentos alinhados ao desempenho da pesquisa. Ademais, pretende-se apresentar o material final diagramado com resultados do estudo.

Além da estratégia de workshops, serão feitas publicações científicas em revistas indexadas, para que haja a devida internacionalização dos resultados do presente estudo.

10. REFERÊNCIAS

1. O'Donnell MJ, Chin SL, Rangarajan S, Xavier D, Liu L, Zhang H, et al. Global and regional effects of potentially modifiable risk factors associated with acute stroke in 32 countries (INTERSTROKE): a case-control study. Lancet (London, England). 2016 Aug;388(10046):761–75.
2. Yusuf S, Hawken S, Ôunpuu S, Dans T, Avezum A, Lanas F, et al. Effect of potentially modifiable risk factors associated with myocardial infarction in 52 countries (the INTERHEARTstudy): case-control study. Lancet [Internet]. 2004 Sep 11;364(9438):937–52. Available from: https://doi.org/10.1016/S0140-6736(04)17018-9
3. Sociedade Brasileira de Genética Médica e Genômica. PARECER TÉCNICO DA SOCIEDADE BRASILEIRA DE GENÉTICA MÉDICA E GENÔMICA SOBRE TESTES GENÉTICOS [Internet]. 2020 [cited 2021 Jul 8]. Available from: https://www.sbgm.org.br/uploads/PARECER TÉCNICO DA SOCIEDADE BRASILEIRA DE GENÉTICA MÉDICA E GENÔMICA SOBRE TESTES GENÉTICOS FINAL.pdf
4. Stram DO. Design, Analysis, and Interpretation of Genome-Wide Association Scans. Springer;

• Rua Treze de Maio, 1.815 • Paulista
• CEP 01327 001 • São Paulo • SP
• Fone: 11 3549 1976 • Fax: 11 3549 0394

2014.

5. Gauderman WJ MJ. Quanto 1.1: A computer program for power and sample size calculations for genetic-epidemiology studies. 2006.

6. BRASIL. Ministério da Saúde. DATASUS [Internet]. Brasília, DF.: Ministério da Saúde; 2016. Available from: datasus.gov.br

7. World Health Organization (WHO). Noncommunicable diseases country profiles 2018 [Internet]. Noncommunicable diseases and mental health. 2018 [cited 2021 May 13]. Available from: https://www.who.int/nmh/publications/ncd-profiles-2018/en/

8. Yusuf S, Joseph P, Rangarajan S, Islam S, Mente A, Hystad P, et al. Modifiable risk factors, cardiovascular disease, and mortality in 155 722 individuals from 21 high-income, middle-income, and low-income countries (PURE): a prospective cohort study. Lancet (London, England). 2020 Mar;395(10226):795–808.

9. Roberts R, Chang CC, Hadley T. Genetic Risk Stratification: A Paradigm Shift in Prevention of Coronary Artery Disease. JACC Basic to Transl Sci [Internet]. 2021;6(3):287–304. Available from: https://www.sciencedirect.com/science/article/pii/S2452302X20303983

10. Lu H, Zhang J, Chen YE, Garcia-Barrio MT. Integration of Transformative Platforms for the Discovery of Causative Genes in Cardiovascular Diseases. Cardiovasc drugs Ther. 2021 Jun;35(3):637–54.

11. Vrablik M, Dlouha D, Todorovova V, Stefler D, Hubacek JA. Genetics of Cardiovascular Disease: How Far Are We from Personalized CVD Risk Prediction and Management? Int J Mol Sci [Internet]. 2021 Apr 17;22(8):4182. Available from: https://pubmed.ncbi.nlm.nih.gov/33920733

12. Lanas F, Avezum A, Bautista LE, Diaz R, Luna M, Islam S, et al. Risk factors for acute myocardial infarction in Latin America: the INTERHEART Latin American study. Circulation. 2007 Mar;115(9):1067–74.

13. Lanas F, Serón P, Lanas A. Coronary heart disease and risk factors in latin america. Glob Heart. 2013 Dec;8(4):341–8.

14. Piegas LS, Avezum A, Pereira JCR, Neto JMR, Hoepfner C, Farran JA, et al. Risk factors for myocardial infarction in Brazil. Am Heart J. 2003 Aug;146(2):331–8.

15. Teo KK, Ounpuu S, Hawken S, Pandey MR, Valentin V, Hunt D, et al. Tobacco use and risk of myocardial infarction in 52 countries in the INTERHEART study: a case-control study. Lancet (London, England). 2006 Aug;368(9536):647–58.

16. Gerstein HC, Islam S, Anand S, Almahmeed W, Damasceno A, Dans A, et al. Dysglycaemia and

• Rua Treze de Maio, 1.815 • Paulista
• CEP 01327 001 • São Paulo • SP
• Fone: 11 3549 1976 • Fax: 11 3549 0394

the risk of acute myocardial infarction in multiple ethnic groups: an analysis of 15,780 patients from the INTERHEART study. Diabetologia. 2010 Dec;53(12):2509–17.

17. Schmidt MI, Duncan BB, Azevedo e Silva G, Menezes AM, Monteiro CA, Barreto SM, et al. Chronic non-communicable diseases in Brazil: burden and current challenges. Lancet (London, England). 2011 Jun;377(9781):1949–61.

18. Pozzan, Roberto; Brandão, Andréa Araújo; Magalhães, Maria Eliane; Freitas, Elisabete Viana de; Brandão AP. O controle da pressão arterial como questão central no tratamento da hipertensão arterial / Blood pressure control as a key factor in the treatment of hypertension. Rev bras Hipertens [Internet]. 2003;10(4):253–9. Available from: https://pesquisa.bvsalud.org/portal/resource/pt/lil-360847?lang=fr

19. Pinho N de A, Pierin AMG. O controle da hipertensão arterial em publicações brasileiras . Vol. 101, Arquivos Brasileiros de Cardiologia . scielo ; 2013. p. e65–73.

20. Walldius G, Jungner I, Holme I, Aastveit AH, Kolar W, Steiner E. High apolipoprotein B, low apolipoprotein A-I, and improvement in the prediction of fatal myocardial infarction (AMORIS study): a prospective study. Lancet [Internet]. 2001 Dec 15;358(9298):2026–33. Available from: https://doi.org/10.1016/S0140-6736(01)07098-2

21. Iqbal R, Anand S, Ounpuu S, Islam S, Zhang X, Rangarajan S, et al. Dietary patterns and the risk of acute myocardial infarction in 52 countries: results of the INTERHEART study. Circulation. 2008 Nov;118(19):1929–37.

22. Leon DA, Saburova L, Tomkins S, Andreev E, Kiryanov N, McKee M, et al. Hazardous alcohol drinking and premature mortality in Russia: a population based case-control study. Lancet (London, England). 2007 Jun;369(9578):2001–9.

23. Leong DP, Smyth A, Teo KK, McKee M, Rangarajan S, Pais P, et al. Patterns of alcohol consumption and myocardial infarction risk: observations from 52 countries in the INTERHEART case-control study. Circulation. 2014 Jul;130(5):390–8.

24. Rosengren A, Hawken S, Ounpuu S, Sliwa K, Zubaid M, Almahmeed WA, et al. Association of psychosocial risk factors with risk of acute myocardial infarction in 11119 cases and 13648 controls from 52 countries (the INTERHEART study): case-control study. Lancet (London, England). 2004 Sep;364(9438):953–62.

25. Gladding PA, Legget M, Fatkin D, Larsen P, Doughty R. Polygenic Risk Scores in Coronary Artery Disease and Atrial Fibrillation. Hear Lung Circ [Internet]. 2020;29(4):634–40. Available from: https://www.sciencedirect.com/science/article/pii/S1443950619315549

26. van Rheenen W, Peyrot WJ, Schork AJ, Lee SH, Wray NR. Genetic correlations of polygenic

• Rua Treze de Maio, 1.815 • Paulista
• CEP 01327 001 • São Paulo • SP
• Fone: 11 3549 1976 • Fax: 11 3549 0394

disease traits: from theory to practice. Nat Rev Genet [Internet]. 2019;20(10):567–81. Available from: https://doi.org/10.1038/s41576-019-0137-z

27. Li R, Chen Y, Ritchie MD, Moore JH. Electronic health records and polygenic risk scores for predicting disease risk. Nat Rev Genet [Internet]. 2020;21(8):493–502. Available from: https://doi.org/10.1038/s41576-020-0224-1

28. Anand SS, Xie C, Paré G, Montpetit A, Rangarajan S, McQueen MJ, et al. Genetic variants associated with myocardial infarction risk factors in over 8000 individuals from five ethnic groups: The INTERHEART Genetics Study. Circ Cardiovasc Genet. 2009 Feb;2(1):16–25.

29. Moura RR de, Coelho AVC, Balbino V de Q, Crovella S, Brandão LAC. Meta-analysis of Brazilian genetic admixture and comparison with other Latin America countries. Am J Hum Biol Off J Hum Biol Counc. 2015;27(5):674–80.

30. Pena SDJ, Santos FR, Tarazona-Santos E. Genetic admixture in Brazil. Am J Med Genet C Semin Med Genet. 2020 Dec;184(4):928–38.

31. Kehdy FSG, Gouveia MH, Machado M, Magalhães WCS, Horimoto AR, Horta BL, et al. Origin and dynamics of admixture in Brazilians and its effect on the pattern of deleterious mutations. Proc Natl Acad Sci [Internet]. 2015 Jul 14;112(28):8696–701. Available from: http://www.pnas.org/lookup/doi/10.1073/pnas.1504447112

32. Carneiro-Proietti ABF, Kelly S, Miranda Teixeira C, Sabino EC, Alencar CS, Capuani L, et al. Clinical and genetic ancestry profile of a large multi-centre sickle cell disease cohort in Brazil. Br J Haematol [Internet]. 2018 Sep;182(6):895–908. Available from: https://onlinelibrary.wiley.com/doi/10.1111/bjh.15462

33. Cubillos-Angulo JM, Arriaga MB, Melo MGM, Silva EC, Alvarado-Arnez LE, de Almeida AS, et al. Polymorphisms in interferon pathway genes and risk of Mycobacterium tuberculosis infection in contacts of tuberculosis cases in Brazil. Int J Infect Dis [Internet]. 2020 Mar;92:21–8. Available from: https://linkinghub.elsevier.com/retrieve/pii/S1201971219304862

34. Kim V, Wal T van der, Nishi MY, Montenegro LR, Carrilho FJ, Hoshida Y, et al. Brazilian cohort and genes encoding for drug-metabolizing enzymes and drug transporters. Pharmacogenomics [Internet]. 2020 Jun;21(9):575–86. Available from: https://www.futuremedicine.com/doi/10.2217/PRS-2020-0013

35. Colares VS, Titan SM de O, Pereira A da C, Malafronte P, Cardena MM, Santos S, et al. MYH9 and APOL1 Gene Polymorphisms and the Risk of CKD in Patients with Lupus Nephritis from an Admixture Population. Assassi S, editor. PLoS One [Internet]. 2014 Mar 21;9(3):e87716. Available from: https://dx.plos.org/10.1371/journal.pone.0087716

• Rua Treze de Maio, 1.815 • Paulista
• CEP 01327 001 • São Paulo • SP
• Fone: 11 3549 1976 • Fax: 11 3549 0394

36. Nunes K, Aguiar VRC, Silva M, Sena AC, de Oliveira DCM, Dinardo CL, et al. How Ancestry Influences the Chances of Finding Unrelated Donors: An Investigation in Admixed Brazilians. Front Immunol [Internet]. 2020 Nov 6;11. Available from: https://www.frontiersin.org/articles/10.3389/fimmu.2020.584950/full
37. Castellucci LC, Almeida L, Cherlin S, Fakiola M, Francis RW, Carvalho EM, et al. A Genome-wide Association Study Identifies SERPINB10, CRLF3, STX7 , LAMP3, IFNG-AS1 , and KRT80 As Risk Loci Contributing to Cutaneous Leishmaniasis in Brazil. Clin Infect Dis [Internet]. 2021 May 18;72(10):e515–25. Available from: https://academic.oup.com/cid/article/72/10/e515/5896307
38. Ribeiro ALP, Duncan BB, Brant LCC, Lotufo PA, Mill JG, Barreto SM. Cardiovascular Health in Brazil. Circulation [Internet]. 2016 Jan 26;133(4):422–33. Available from: https://www.ahajournals.org/doi/10.1161/CIRCULATIONAHA.114.008727
39. Assimes TL, Roberts R. Genetics: Implications for Prevention and Management of Coronary Artery Disease. J Am Coll Cardiol. 2016 Dec;68(25):2797–818.
40. Larsson SC, Burgess S, Michaëlsson K. Association of Genetic Variants Related to Serum Calcium Levels With Coronary Artery Disease and Myocardial Infarction. JAMA. 2017 Jul;318(4):371–80.
41. Wang J, Xu D, Wu X, Zhou C, Wang H, Guo Y, et al. Polymorphisms of matrix metalloproteinases in myocardial infarction: a meta-analysis. Heart. 2011 Oct;97(19):1542–6.
42. Lechner K, Kessler T, Schunkert H. Should We Use Genetic Scores in the Determination of Treatment Strategies to Control Dyslipidemias? Curr Cardiol Rep. 2020 Sep;22(11):146.
43. Weale ME, Riveros-Mckay F, Selzam S, Seth P, Moore R, Tarran WA, et al. Validation of an Integrated Risk Tool, Including Polygenic Risk Score, for Atherosclerotic Cardiovascular Disease in Multiple Ethnicities and Ancestries. Am J Cardiol [Internet]. 2021;148:157–64. Available from: https://www.sciencedirect.com/science/article/pii/S0002914921002071
44. Rannikmäe K, Sivakumaran V, Millar H, Malik R, Anderson CD, Chong M, et al. COL4A2 is associated with lacunar ischemic stroke and deep ICH: Meta-analyses among 21,500 cases and 40,600 controls. Neurology. 2017 Oct;89(17):1829–39.
45. Rannikmäe K, Davies G, Thomson PA, Bevan S, Devan WJ, Falcone GJ, et al. Common variation in COL4A1/COL4A2 is associated with sporadic cerebral small vessel disease. Neurology [Internet]. 2015/02/04. 2015 Mar 3;84(9):918–26. Available from: https://pubmed.ncbi.nlm.nih.gov/25653287

• Rua Treze de Maio, 1.815 • Paulista
• CEP 01327 001 • São Paulo • SP
• Fone: 11 3549 1976 • Fax: 11 3549 0394

46. Das S, Kaul S, Jyothy A, Munshi A. Role of TLR4 (C1196T) and CD14 (C-260T) Polymorphisms in Development of Ischemic Stroke, Its Subtypes and Hemorrhagic Stroke. J Mol Neurosci. 2017 Dec;63(3–4):300–7.
47. Xue Y, Zhang L, Fan Y, Li Q, Jiang Y, Shen C. C-Reactive Protein Gene Contributes to the Genetic Susceptibility of Hemorrhagic Stroke in Men: a Case-Control Study in Chinese Han Population. J Mol Neurosci. 2017 Aug;62(3–4):395–401.
48. Chauhan G, Debette S. Genetic Risk Factors for Ischemic and Hemorrhagic Stroke. Curr Cardiol Rep. 2016 Dec;18(12):124.
49. Catchpool M, Ramchand J, Martyn M, Hare DL, James PA, Trainer AH, et al. A cost-effectiveness model of genetic testing and periodical clinical screening for the evaluation of families with dilated cardiomyopathy. Genet Med [Internet]. 2019 Dec;21(12):2815–22. Available from: https://linkinghub.elsevier.com/retrieve/pii/S1098360021012193
50. Ingles J, McGaughran J, Scuffham PA, Atherton J, Semsarian C. A cost-effectiveness model of genetic testing for the evaluation of families with hypertrophic cardiomyopathy. Heart [Internet]. 2012 Apr 15;98(8):625–30. Available from: https://heart.bmj.com/lookup/doi/10.1136/heartjnl-2011-300368
51. Wonderling D, Umans-Eckenhausen M, Marks D, Defesche J, Kastelein J, Thorogood M. Cost-Effectiveness Analysis of the Genetic Screening Program for Familial Hypercholesterolemia in the Netherlands. Semin Vasc Med [Internet]. 2004 Mar 22;4(01):97–104. Available from: http://www.thieme-connect.de/DOI/DOI?10.1055/s-2004-822992
52. Mujwara D, Henno G, Vernon ST, Peng S, Di Domenico P, Schroeder B, et al. Integrating a Polygenic Risk Score for Coronary Artery Disease as a Risk Enhancing Factor in the Pooled Cohort Equation is Cost-effective in a US Health System. medRxiv [Internet]. 2021 Jan 1;2021.06.21.21259210. Available from: http://medrxiv.org/content/early/2021/06/25/2021.06.21.21259210.abstract
53. Sussman J, Marrero W, Burke J, Lavieri M, Hayward RA. Abstract 101: Cost-Effectiveness and Decision Analysis of Polygenic Risk Scores in Statin Use for Primary Prevention. Circ Cardiovasc Qual Outcomes [Internet]. 2018 Apr 1;11(suppl_1):A101–A101. Available from: https://doi.org/10.1161/circoutcomes.11.suppl_1.101
54. Kiflen M. Cost-Utility Analysis of Using Polygenic Risk Scores to Guide Statin Therapy for Cardiovascular Disease. Mc Master; 2020.
55. Adeyemo A, Balaconis MK, Darnes DR, Fatumo S, Granados Moreno P, Hodonsky CJ, et al. Responsible use of polygenic risk scores in the clinic: potential benefits, risks and gaps. Nat

• Rua Treze de Maio, 1.815 • Paulista
• CEP 01327 001 • São Paulo • SP
• Fone: 11 3549 1976 • Fax: 11 3549 0394

Med [Internet]. 2021 Nov 15;27(11):1876–84. Available from: https://www.nature.com/articles/s41591-021-01549-6

56. von Elm E, Altman DG, Egger M, Pocock SJ, Gøtzsche PC, Vandenbroucke JP. The Strengthening the Reporting of Observational Studies in Epidemiology (STROBE) statement: guidelines for reporting observational studies. J Clin Epidemiol. 2008 Apr;61(4):344–9.

57. Bhatt DL. International Prevalence, Recognition, and Treatment of Cardiovascular Risk Factors in Outpatients With Atherothrombosis. JAMA [Internet]. 2006 Jan 11;295(2):180. Available from: http://jama.jamanetwork.com/article.aspx?doi=10.1001/jama.295.2.180

58. Rodrigo LM, Nyholt DR. Imputation and Reanalysis of ExomeChip Data Identifies Novel, Conditional and Joint Genetic Effects on Parkinson's Disease Risk. Genes (Basel) [Internet]. 2021 May 4;12(5):689. Available from: https://www.mdpi.com/2073-4425/12/5/689

59. Danilov KA, Nikogosov DA, Musienko S V., Baranova A V. A comparison of BeadChip and WGS genotyping outputs using partial validation by sanger sequencing. BMC Genomics [Internet]. 2020 Sep 10;21(S7):528. Available from: https://bmcgenomics.biomedcentral.com/articles/10.1186/s12864-020-06919-x

60. Tachmazidou I, Süveges D, Min JL, Ritchie GRS, Steinberg J, Walter K, et al. Whole-Genome Sequencing Coupled to Imputation Discovers Genetic Signals for Anthropometric Traits. Am J Hum Genet [Internet]. 2017 Jun;100(6):865–84. Available from: https://linkinghub.elsevier.com/retrieve/pii/S0002929717301593

61. Höglund J, Rafati N, Rask-Andersen M, Enroth S, Karlsson T, Ek WE, et al. Improved power and precision with whole genome sequencing data in genome-wide association studies of inflammatory biomarkers. Sci Rep [Internet]. 2019 Dec 14;9(1):16844. Available from: http://www.nature.com/articles/s41598-019-53111-7

62. Chen S-F, Dias R, Evans D, Salfati EL, Liu S, Wineinger NE, et al. Genotype imputation and variability in polygenic risk score estimation. Genome Med [Internet]. 2020 Dec 23;12(1):100. Available from: https://genomemedicine.biomedcentral.com/articles/10.1186/s13073-020-00801-x

63. De La Vega FM, Bustamante CD. Polygenic risk scores: a biased prediction? Genome Med [Internet]. 2018 Dec 27;10(1):100. Available from: https://genomemedicine.biomedcentral.com/articles/10.1186/s13073-018-0610-x

64. Hadley TD, Agha AM, Ballantyne CM. How Do We Incorporate Polygenic Risk Scores in Cardiovascular Disease Risk Assessment and Management? Curr Atheroscler Rep [Internet]. 2021 Jun 1;23(6):28. Available from: https://link.springer.com/10.1007/s11883-021-00915-

• Rua Treze de Maio, 1.815 • Paulista
• CEP 01327 001 • São Paulo • SP
• Fone: 11 3549 1976 • Fax: 11 3549 0394

6
65. Homburger JR, Neben CL, Mishne G, Zhou AY, Kathiresan S, Khera A V. Low coverage whole genome sequencing enables accurate assessment of common variants and calculation of genome-wide polygenic scores. Genome Med [Internet]. 2019 Dec 26;11(1):74. Available from: https://genomemedicine.biomedcentral.com/articles/10.1186/s13073-019-0682-2
66. Li JH, Mazur CA, Berisa T, Pickrell JK. Low-pass sequencing increases the power of GWAS and decreases measurement error of polygenic risk scores compared to genotyping arrays. Genome Res [Internet]. 2021 Apr;31(4):529–37. Available from: http://genome.cshlp.org/lookup/doi/10.1101/gr.266486.120
67. Rubinacci S, Ribeiro DM, Hofmeister RJ, Delaneau O. Efficient phasing and imputation of low-coverage sequencing data using large reference panels. Nat Genet [Internet]. 2021 Jan 7;53(1):120–6. Available from: http://www.nature.com/articles/s41588-020-00756-0
68. Lewis CM, Vassos E. Polygenic risk scores: from research tools to clinical instruments. Genome Med [Internet]. 2020;12(1):44. Available from: https://doi.org/10.1186/s13073-020-00742-5
69. Babb de Villiers C, Kroese M, Moorthie S. Understanding polygenic models, their development and the potential application of polygenic scores in healthcare. J Med Genet. 2020 Nov;57(11):725–32.
70. European Molecular Biology Laboratory. PSG Catalog [Internet]. PSG catalog site. 2021 [cited 2021 Oct 25]. Available from: https://www.PRScatalog.org/
71. Buniello A, MacArthur JAL, Cerezo M, Harris LW, Hayhurst J, Malangone C, et al. The NHGRI-EBI GWAS Catalog of published genome-wide association studies, targeted arrays and summary statistics 2019. Nucleic Acids Res [Internet]. 2019 Jan 8;47(D1):D1005–12. Available from: https://academic.oup.com/nar/article/47/D1/D1005/5184712
72. Novembre DHASSSJ, Lange K. Admixture 1.3 Software Manual [Internet]. 2015 [cited 2021 Oct 25]. Available from: https://vcru.wisc.edu/simonlab/bioinformatics/programs/admixture/admixture-manual.pdf
73. Santos HC, Horimoto AVR, Tarazona-Santos E, Rodrigues-Soares F, Barreto ML, Horta BL, et al. A minimum set of ancestry informative markers for determining admixture proportions in a mixed American population: the Brazilian set. Eur J Hum Genet [Internet]. 2016 May 23;24(5):725–31. Available from: http://www.nature.com/articles/ejhg2015187
74. Choi SW, Mak TS-H, O'Reilly PF. Tutorial: a guide to performing polygenic risk score analyses.

• Rua Treze de Maio, 1.815 • Paulista
• CEP 01327 001 • São Paulo • SP
• Fone: 11 3549 1976 • Fax: 11 3549 0394

Nat Protoc [Internet]. 2020;15(9):2759–72. Available from: https://doi.org/10.1038/s41596-020-0353-1
75. Chang CC, Chow CC, Tellier LC, Vattikuti S, Purcell SM, Lee JJ. Second-generation PLINK: rising to the challenge of larger and richer datasets. Gigascience. 2015;4:7.
76. Purcell S, Neale B, Todd-Brown K, Thomas L, Ferreira MAR, Bender D, et al. PLINK: a tool set for whole-genome association and population-based linkage analyses. Am J Hum Genet [Internet]. 2007/07/25. 2007 Sep;81(3):559–75. Available from: https://pubmed.ncbi.nlm.nih.gov/17701901
77. Privé F, Vilhjálmsson BJ, Aschard H, Blum MGB. Making the Most of Clumping and Thresholding for Polygenic Scores. Am J Hum Genet [Internet]. 2019;105(6):1213–21. Available from: https://www.sciencedirect.com/science/article/pii/S0002929719304227
78. Mak TSH, Porsch RM, Choi SW, Zhou X, Sham PC. Polygenic scores via penalized regression on summary statistics. Genet Epidemiol. 2017 Sep;41(6):469–80.
79. Privé F, Arbel J, Vilhjálmsson BJ. LDpred2: better, faster, stronger. Bioinformatics [Internet]. 2020 Dec 1;36(22–23):5424–31. Available from: https://doi.org/10.1093/bioinformatics/btaa1029
80. Privé F, Aschard H, Ziyatdinov A, Blum MGB. Efficient analysis of large-scale genome-wide data with two R packages: bigstatsr and bigsnpr. Bioinformatics. 2018 Aug;34(16):2781–7.
81. Khera A V, Chaffin M, Aragam KG, Haas ME, Roselli C, Choi SH, et al. Genome-wide polygenic scores for common diseases identify individuals with risk equivalent to monogenic mutations. Nat Genet [Internet]. 2018;50(9):1219–24. Available from: https://doi.org/10.1038/s41588-018-0183-z
82. Márquez-Luna C, Loh P-R, Price AL. Multiethnic polygenic risk scores improve risk prediction in diverse populations. Genet Epidemiol. 2017 Dec;41(8):811–23.
83. Khera A V., Kathiresan S. Genetics of coronary artery disease: discovery, biology and clinical translation. Nat Rev Genet [Internet]. 2017 Jun 13;18(6):331–44. Available from: http://www.nature.com/articles/nrg.2016.160
84. Beheshti SO, Madsen CM, Varbo A, Nordestgaard BG. Worldwide Prevalence of Familial Hypercholesterolemia. J Am Coll Cardiol [Internet]. 2020 May;75(20):2553–66. Available from: https://linkinghub.elsevier.com/retrieve/pii/S0735109720347501
85. Khera A V., Chaffin M, Zekavat SM, Collins RL, Roselli C, Natarajan P, et al. Whole-Genome Sequencing to Characterize Monogenic and Polygenic Contributions in Patients Hospitalized With Early-Onset Myocardial Infarction. Circulation [Internet]. 2019 Mar 26;139(13):1593–

• Rua Treze de Maio, 1.815 • Paulista
• CEP 01327 001 • São Paulo • SP
• Fone: 11 3549 1976 • Fax: 11 3549 0394

602. Available from: https://www.ahajournals.org/doi/10.1161/CIRCULATIONAHA.118.035658

86. Berberich AJ, Hegele RA. The complex molecular genetics of familial hypercholesterolaemia. Nat Rev Cardiol [Internet]. 2019 Jan 4;16(1):9–20. Available from: http://www.nature.com/articles/s41569-018-0052-6

87. Watkins H, Farrall M. Genetic susceptibility to coronary artery disease: from promise to progress. Nat Rev Genet [Internet]. 2006 Mar 7;7(3):163–73. Available from: http://www.nature.com/articles/nrg1805

88. Pearce N. Analysis of matched case-control studies. BMJ [Internet]. 2016 Feb 25;i969. Available from: https://www.bmj.com/lookup/doi/10.1136/bmj.i969

89. Joseph PG, Pare G, Asma S, Engert JC, Yusuf S, Anand SS. Impact of a Genetic Risk Score on Myocardial Infarction Risk Across Different Ethnic Populations. Can J Cardiol [Internet]. 2016 Dec;32(12):1440–6. Available from: https://linkinghub.elsevier.com/retrieve/pii/S0828282X1630126X

90. Stram DO. Design, Analysis, and Interpretation of Genome-Wide Association Scans [Internet]. New York, NY: Springer New York; 2014. (Statistics for Biology and Health). Available from: http://link.springer.com/10.1007/978-1-4614-9443-0

91. Joseph M. Hilbe. Practical Guide to Logistic Regression. 2015.

92. David W. Hosmer Jr., Stanley Lemeshow RXS. Applied Logistic Regression. 3rd ed. 2013.

93. Ørnulf Borgan, Norman Breslow, Nilanjan Chatterjee, Mitchell H. Gail, Alastair Scott CJW. Handbook of Statistical Methods for Case-Control Studies. 2018.

94. Liu Y, Nickleach DC, Zhang C, Switchenko JM, Kowalski J. Carrying out streamlined routine data analyses with reports for observational studies: introduction to a series of generic SAS® macros. F1000Research [Internet]. 2019 Jun 5;7:1955. Available from: https://f1000research.com/articles/7-1955/v2

95. Bursac Z, Gauss CH, Williams DK, Hosmer DW. Purposeful selection of variables in logistic regression. Source Code Biol Med [Internet]. 2008 Dec 16;3(1):17. Available from: https://scfbm.biomedcentral.com/articles/10.1186/1751-0473-3-17

96. Bursac ZG, Williams D HD. A Purposeful Selection of Variables Macro for Logistic Regression. In: SAS Global Forum 2007 Conference. 2007.

97. Patrick Royston WS. Multivariable Model - Building: A Pragmatic Approach to Regression Anaylsis based on Fractional Polynomials for Modelling Continuous Variables. 2008.

98. Mithat Gönen. Analyzing Receiver Operating Characteristic Curves with SAS. SAS Institute;

• Rua Treze de Maio, 1.815 • Paulista
• CEP 01327 001 • São Paulo • SP
• Fone: 11 3549 1976 • Fax: 11 3549 0394

2007.

99. Allison PD. Logistic regression using SAS: Theory and application. 2ng ed. SAS Institute; 2012.

100. Glenn A. Walker and Jack Shostak. Common Statistical Methods for Clinical Research with SAS Examples. 3rd ed. SAS Institute; 2010.

101. Lang TA SM. How to Report Statistics in Medicine: Annotated Guidelines for Authors, Editors,and Reviewers. 2nd ed. American College of Physicians; 2006.

102. Romão X, Delgado R, Costa A. An empirical power comparison of univariate goodness-of-fit tests for normality. J Stat Comput Simul [Internet]. 2010 May;80(5):545–91. Available from: http://www.tandfonline.com/doi/abs/10.1080/00949650902740824

103. Yap BW, Sim CH. Comparisons of various types of normality tests. J Stat Comput Simul [Internet]. 2011 Dec;81(12):2141–55. Available from: http://www.tandfonline.com/doi/abs/10.1080/00949655.2010.520163

• Rua Treze de Maio, 1.815 • Paulista
• CEP 01327 001 • São Paulo • SP
• Fone: 11 3549 1976 • Fax: 11 3549 0394

PROTOCOL – English version

A- General Objective

To assess the population attributable risk associated with the presence of polymorphisms in genes related to cardiovascular diseases and their impact as an independent risk factor for the occurrence of AMI, stroke and thrombotic-ischemic events in peripheral arterial territory in the Brazilian population.

B- Specific Objectives

- To perform genotyping of patients with and without overt cardiovascular disease, analyzing polymorphisms associated with cardiovascular diseases;
- To assess the impact of genetic polymorphisms as an independent risk factor for atherosclerotic cardiovascular disease;
- To assess the correlation between genetic findings and phenotypic factors in the occurrence of atherosclerotic CVD;
- To establish the genomic profile associated with the occurrence of atherosclerotic CVD, considering the extensive miscegenation of the Brazilian population and limited data and lacking robustness for a small number of individuals evaluated in other studies.

RATIONALE

Cardiovascular diseases are responsible for more than 300,000 deaths a year, representing the main cause of death in Brazil, followed by neoplasms, respiratory diseases, and diabetes. Together, chronic non-communicable diseases (NCDs) are responsible for approximately 70% of the causes of death in both sexes (6,7).
Currently, it is known that seven out of ten cases of cardiovascular disease can be explained by risk factors such as high blood pressure, low education, smoking, dyslipidemia, inadequate diet, abdominal obesity, lack of regular physical activity, diabetes, psychosocial changes, and air pollution - all of which can be modified (8). However, in recent years, advances in genetic sequencing technologies associated with greater accessibility to them due to cost reduction, led to the identification of genetic polymorphisms associated with increased risk of cardiovascular diseases, reinforcing the model of genetic interaction and the environment as a trigger for cardiovascular diseases, which, unlike other known risk factors, is a non-modifiable factor (9–11).

RISK FACTORS

It is estimated that about 90% of cases of AMI and CVA are associated with traditional risk factors and population aging, smoking, inadequate diet and sedentary lifestyle are associated with the increasing prevalence of these factors. At the national level, we have limitations of representative data in terms of epidemiological surveillance for NCDs and determinant risk factors (6,8,12).
INTERHEART, a case-control study that evaluated the effects of potentially modifiable risk factors associated with AMI in 52 countries and involved 30,000 individuals (15,152 cases and 14,820 controls), identified that smoking, diabetes, high blood pressure, obesity, anxiety, and depression, inadequate diet, physical inactivity, alcohol consumption, and apolipoprotein B/A1 ratio (ApoB/ApoA1) constitute the most important risk factors associated with first AMI in all geographic regions, in men and women and with representative ethnic diversity (2).
In Latin America, the analysis of the results coincided with those observed by global INTERHEART, demonstrating special importance for abdominal obesity, dyslipidemia, smoking, and hypertension. These results coincide with a

• Rua Treze de Maio, 1.815 • Paulista
• CEP 01327 001 • São Paulo • SP
• Fone: 11 3549 1976 • Fax: 11 3549 0394

previously published study, where risk factors were evaluated only in the Brazilian population (12–14).

Smoking is associated with several diseases and is responsible for 50% of preventable deaths in smokers, half of which are due to CVD. It is estimated that half of smoking deaths occur in individuals between 35-69 years of age, who lose an average of 22 years of life due to the habit (13,15). In Brazil, the prevalence is still at 15.5%. In all countries, the habit of smoking is more prevalent in men. However, the difference in this proportion has been decreasing and represents an important risk factor in women, with a linear relationship according to the number of cigarettes/day (15).

The prevalence of diabetes in adults has been progressively increasing, with a substantial contribution from changes in diet and obesity. Mortality by gender and age is 58% higher in individuals with diabetes compared to non-diabetic controls. Of all deaths in diabetic patients, 38% are due to CVD (12,13,16,17). The presence of diabetes increases the cardiovascular risk by 2-3 times. INTERHEART, for example, revealed 4.2 times more chances for the occurrence of AMI in diabetics in the Brazilian population. There is even a higher risk of AMI in women with diabetes (2). Glycated hemoglobin (HBA1C) values > 5.4% are associated with a 22% higher cardiovascular risk compared to individuals with lower values.

High blood pressure (SAH) is an independent and most prevalent factor for cardiovascular risk. It is estimated that the prevalence of SAH in Latin America is 29.1% and in Brazil it is approximately 24%. When evaluating individuals over 60 years of age, this prevalence rises to approximately 50%. Regarding CAD, treatment with anti-hypertensive drugs reduces the risk by 20-25% (18). However, publications describe rates of arterial hypertension control in Brazil between 10.1% and 52.4%. Considering an optimistic estimate, about 50% of treated hypertensive patients would be exposed to the complications of the disease due to uncontrolled blood pressure (18,19).

The elevation of LDL is one of the most important factors for CAD, but its values are influenced by the acute phase of AMI or lack of adequate fasting to measure its levels. ApoB is not influenced by the level of fasting and reflects the amount of potentially atherogenic lipoproteins, and ApoA-1 represents high-density antiatherogenic lipoproteins. Some studies show that the ApoB/ApoA ratio is a better predictor of AMI than the LDL/HDL ratio, as observed in the AMORIS study (2,20). It is estimated that 25% of the Brazilian population has total cholesterol above 190 mg/dl, and in individuals over 45 years of age this proportion rises to 33%.

Obesity, a morbidity defined as values of Body Mass Index (BMI) above 30 kg/m^2, presents an increasing expansion in its prevalence, also behaving as an independent risk factor for AMI (10). Currently, it is estimated that 14.8% are obese in the population over 20 years of age. Obesity is also a reflection of an inadequate diet and sedentary lifestyle, habits that are increasingly common in Brazil and worldwide. The INTERHEART study in Latin America showed that a high waist-to-hip ratio is associated with a 2.5 times greater chance of AMI in men and 4.1 times in women (13,14). INTERHEART Latin America also showed a clear inverse association between consumption of a diet rich in fruits and vegetables and the occurrence of AMI. A positive association was also observed between the consumption of fried foods and salty snacks and the occurrence of AMI (14,21). Regarding physical inactivity, data show that only 14.7% of the Brazilian population practice regular physical activities, with only 3.3% performing the 30 minutes/day, five times/week recommended by the WHO (14,17). Regular physical activity can be responsible for a 40% reduction in the relative risk for the occurrence of the first AMI. However, the proportion of physically active individuals in this population is still very low (2).

• Rua Treze de Maio, 1.815 • Paulista
• CEP 01327 001 • São Paulo • SP
• Fone: 11 3549 1976 • Fax: 11 3549 0394

In 2010, alcohol consumption was responsible for 2.5 million deaths worldwide, mainly due to external causes, liver cirrhosis, CVD, and cancer. Additionally, the frequency and consumption pattern can be an important modifier of the association between alcohol consumption and AMI. There is evidence that episodic consumption of high amounts of alcohol is a risk factor for sudden cardiac death (22,23). In Brazil, despite the fact that 45% of individuals are teetotalers, the proportion of excessive consumers is 25%.

In Brazil, it is estimated that 18.8% of individuals have already been diagnosed with depression and, when the evaluation criterion includes depressive and anxiety disorders, this number reaches 30% (14). The INTERHEART study demonstrated that the effect of stress is independent of socioeconomic profile and smoking and consistent across geographic regions, age and gender assessed, and independently after adjustment for covariates (24). In Latin America, only a slight association between depression and risk of AMI was observed. In Brazil, an odds ratio of 1.48 (95% CI 1.0-2.22) was observed.

GENETIC FACTORS AND CARDIOVASCULAR DISEASE

In the study of the contribution of the genetic background in the pathogenesis of diseases, the main forms of presentation include mutation in genes with a potential direct effect of disease manifestation (monogenic diseases) or genes that present polymorphisms/mutations (polygenic diseases) that behave as susceptibility factors for a disease, in the "two hits" pathogenic model, where there is a need for a genetic background associated with environmental factors for the development of the disease (25–27).

Some studies have evaluated the potential impact of polymorphisms on cardiovascular disease. A sub-analysis of the INTERHEART study, the INTERHEART Genetics Study, in a case-control study design evaluated the impact of 1,536 single-nucleotide polymorphisms (SNPs) on 103 genes in a multiethnic population of 8,034 patients. The objective was to assess the impact of genetic polymorphisms associated with cardiovascular disease on the risk of AMI. When evaluated with other cardiovascular risk factors (APO B/A, hypertension, diabetes, abdominal obesity, smoking, physical inactivity, alcoholism and depression), thirteen polymorphisms were associated with increased cardiovascular risk, 11 of which related to serum levels of APO B/A, 1 associated with LDL receptor, and 1 with Apolipoprotein E (28). However, as already mentioned, there are no data exclusively from Brazilian individuals with adequate statistical power and with systematization of obtaining information on traditional risk factors, essential aspects and, thus, recommended for an integrated analysis with the objective of evaluating the contribution of the genetic risk score on the impact on the population attributable risk for the occurrence of atherosclerotic events in its wide clinical spectrum.

The Brazilian population, due to its ethnic diversity, miscegenation, multiple ancestry, and high prevalence of consanguineous marriages, has a unique genetic peculiarity that may reflect on the predisposition to diseases, including cardiovascular diseases (29,30). The interference of the genetic component on the pattern or susceptibility to diseases in the Brazilian population has already been shown to have an impact on the control and treatment of infectious, autoimmune, hematological diseases, drug pharmacokinetics, and even on the transplant allocation system (31–37). In a review article on cardiovascular health in Brazil (38), several points are described on important issues inherent to the public health system and epidemiological characteristics, in addition to the factors already known associated with CVD. The authors point out the next challenges for a better understanding of CVD in Brazil and mention, among them, the need for robust studies, with national representation, that allow the knowledge of the epidemiological peculiarities and genetic contributors to the genesis of CVD. Thus, we propose to answer this relevant population question through the CV-Genes Study, with correction/adjustment for traditional cardiovascular risk factors, since genetic background is the only non-controllable or modifiable risk factor and knowing its real impact on the Brazilian population may lead to the implementation of population genetic screening programs for strict control of cardiovascular risk factors and/or indicate changes in therapeutic targets and clinical control.

A review (39) of the main studies that evaluated susceptibility loci for coronary artery disease was recently published. This review included studies from important consortia, such as CARDIoGRAM, MIGen, WTCCC, and Cardiogenics, deCODE, CARDIoGRAM, C4D, and CARDIoGRAM + C4D, which together contributed to the identification of 60 loci, whose mechanism of action is related to serum levels of LDL, lipoprotein A and triglycerides, blood pressure, body mass index, coagulation profile, changes in endothelial and smooth muscle cells of the vascular wall, mechanisms of cell migration and adhesion, immune activation, inflammation, cell growth, differentiation and apoptosis, as well as constituents of the extracellular matrix and also some loci with unknown function. Table 1, extracted from the article in question, lists the potential loci, potential mechanisms of action and from which consortium such information was acquired.

Polymorphisms in genes associated with the regulation of the calcium (*CASR, CYP24A1, CARS, DGKD, DGKH/KIAA0564*, and *GATA3*) (40) metalloproteinases (MMP-3 and MMP-9) levels (41) were also associated with an increased risk of coronary artery disease and AIM. The list of polymorphisms associated with AMI progressively increases, and some authors have already reinforced the importance of using the genetic background in cardiovascular risk calculators (9,42,43).

Regarding cases of stroke, polymorphisms in some genes, such as *MTHFR, eNOS, ACE, AGT, ApoE, PON1, PDE4D*, were associated with a higher risk of ischemic stroke. Expectedly, there were polymorphisms in some genes. For cases of hemorrhagic stroke, polymorphisms in collagen (44,45), TLR4 and CD14 (46) and even in the gene that gives rise to C-reactive protein were identified (47). Table 2 and Figure 2 provide a summary of polymorphisms associated with increased risk of ischemic and hemorrhagic stroke.

• Rua Treze de Maio, 1.815 • Paulista
• CEP 01327 001 • São Paulo • SP
• Fone: 11 3549 1976 • Fax: 11 3549 0394

The description of the methodology of this case-control study will follow the items established by the reporting tool The Strengthening the Reporting of Observational Studies in Epidemiology (STROBE) (49).

Study design

This is a cardiovascular case-control study with the incorporation of genetic data to assess the impact of genomic information, previously associated with polygenic cardiovascular diseases (CVD), as an independent risk predictor (expressed in Odds Ratio) and in conjunction with traditional risk factors (smoking, diabetes, high blood pressure, obesity, anxiety and depression, inadequate diet, physical inactivity, alcohol consumption, and apolipoprotein B/A1 ratio (ApoB/ApoA1)).

Background

The study will be carried out in about 50 reference sites for cardiology care, covering the five Brazilian regions. The study will be conducted from January 2022 to December 2023. Data collection will be carried out in each site consecutively, for cases and controls, through electronic CRF, during the period of 02 years for conducting the study.

Participants

Cases (N = 1,867) will be selected by the occurrence of the first atherosclerotic cardiovascular event (Acute Myocardial Infarction, Stroke, and Peripheral Arterial Thrombotic-Ischemic Events) during the hospitalization phase for the management of the acute atherothrombotic event. The ratio between cases and controls will be 1:1. Controls (N = 1,867) will be adult individuals over 18 years of age who sought medical care at the same locations for other clinical reasons (no CVD) or individuals without any overt disease. The definitions of acute atherothrombotic events listed above follow classic clinical and complementary test criteria and are based on national and international guidelines. The complete project will be submitted to the EC/CONEP system, and after ethical approval, all cases and controls will be invited to participate and, if they agree, an Informed Consent Form will be obtained.

Variables and Endpoints

As it is a case-control study, there will be no follow-up for the occurrence of clinical events. Therefore, we will assess exposures to traditional risk factors in combination with genomic data (polygenic risk score) in both cases and controls, and these exposures will be expressed as odds ratios. Multiple logistic regression models will be built to adjust and determine the strengths of association between demographic variables, traditional risk factors, and genetic data: Gender, age, ethnicity, weight, body mass index, smoking, diabetes, high blood pressure, obesity, anxiety and depression, inadequate diet, physical inactivity, alcohol consumption, and apolipoprotein B/A1 ratio (ApoB/ApoA1). For each association variable with a higher chance of cardiovascular disease (significant OR), an attributable risk will be calculated to estimate the fraction of risk attributable to the genetic component (PGS) and other clinical and demographic variables.

Data source and measurement

Data will be collected in an electronic questionnaire (e-CRF), to be developed and made available through the RedCap application. This form will include all the results of clinical and demographic variables (above), in addition to the results of PGS and other laboratory tests (Glycated hemoglobin, serum apolipoprotein A-1, serum apolipoprotein B, serum cholesterol, total serum cholesterol and fractions (HDL, LDL, and VLDL), serum triglycerides, urine creatinine, serum sodium, serum potassium, urine sodium, urine potassium, urine isolated creatinine, urine isolated albuminuria).

The genetic evaluation will be performed through the association of Low-covering Whole Genome Sequencing (coverage 0.5-5x) and Whole Exome Sequencing (average coverage 30x). From this test, the PGS will be available, as described below.

• Rua Treze de Maio, 1.815 • Paulista
• CEP 01327 001 • São Paulo • SP
• Fone: 11 3549 1976 • Fax: 11 3549 0394

DNA EXTRACTION FROM SAMPLES

DNA from blood samples will be extracted by an automated process (QIASymphony, using the QIAmp DNA Mini Kit – Qiagen) following the manufacturers' instructions. The extracted DNA will be analyzed for quantification by the Qubit Fluorometer (Thermo Fisher) and will be kept at -20 °C until use.

CONSTRUCTION OF THE EXOME AND LOW PASS 1x GENOME LIBRARIES AND SEQUENCING

For the construction of the exome and low pass 1x genome libraries, the Twist Bioscience company's preparation protocol will be followed with enzymatic fragmentation (Enzymatic Fragmentation and Combinatorial Dual Indices) using 50 ng of DNA input. The construction of the exome library by hybrid capture probes will take around 26 hours for completion, and for the Low-pass 1x genome, completion will take around 4 hours.

DNA will be sequenced on a NovaSeq platform (IlIumina) using the NovaSeq S4 kit with 300 cycles (2 x 150 bp - paired end). Run data and quality control will be monitored in NovaSeq control software.

ANALYSIS OF THE GENERATED DATA

The generated data will be processed in two distinct pipelines. The Exome pipeline is already deployed and functional. The pipeline that will process the whole genome data (WGS) is under development. Both pipelines are the responsibility of the Data Science and Bioinformatics Group of R&D (Grupo Fleury).

Both pipelines are based on Illumina's Dragen v.3.6.4 system installed on servers located at Fleury Group's headquarters. At the end of the runs, the pipeline will perform the demultiplexing step, creating the FASTQ files (containing the reads) for each sample, aligning the reads to the reference human genome (Hg19), creating the BAM files containing the aligned reads and creating the VCFs files containing the identified variants (SNPs, INDELS, CNV). After completing this step, the generated BAM and VCF files will be stored in the cloud (AWS) and then sent to the Emedgene company platform for annotation/classification of the variants found in the genes: LDLR, APOB, PCSK9, LDLRAP1, ABCG5, ABCG8, APOE, LIPA. All data generated will be processed, analyzed, and reported by specialized professionals (bioinformaticians, analysts, and geneticists). Low-pass 1x genome data will be submitted to the imputation method, which guarantees quality in the identification of variants and will be used with the call of exome variants by the systems and data team for PRS calibration.

CALIBRATION AND CALCULATION OF PGS FOR HEART DISEASES

The literature on polygenic score (PGS) calculation is relatively recent. Despite this, its evolution grows at an accelerated pace, mainly due to the increase in genetic datasets that allow the proposition of new calculation methods and the growth of its use for disease prediction, as a result of the increase in its accuracy in research based on cases control and population in general (50).

PGS aggregates the effects of genetic variants into a single number that predicts genetic predisposition to a phenotype. PGS are typically composed of hundreds to millions of genetic variants (usually SNPs). For each individual, the number of risk alleles computed in each variant is summed and weighed by the estimated value of the effects obtained (log odds ratio for traits with binary values or Beta coefficients for traits with continuous value (50)) from large-scale genomic studies (GWAS), as can be seen in equation 1:

$$PGS= \beta_i k_i+ \beta_{(i+1)} k_{(i+1)}+\cdots +\beta_n k_n \qquad (1)$$

where $\beta_i,\beta_{(i+1)},\ldots,\beta_n$ are the effects obtained, $k_i,k_{(i+1)},\ldots,k_n$ the number of risk alleles computed in each variant (0, 1 or 2), and $i,i+1,\ldots,n$ are the indices of the SNPs (51).

SELECTION AND EVALUATION OF PGS MODELS

• Rua Treze de Maio, 1.815 • Paulista
• CEP 01327 001 • São Paulo • SP
• Fone: 11 3549 1976 • Fax: 11 3549 0394

For the calculation and calibration of PGS for heart diseases, previous PGS models will be selected from the PGS Catalog (52). The PGS Catalog is an open database of published polygenic scores (PGS). Each PGS is consistently annotated with relevant metadata including score files, which contain information on variants, risk alleles (effect allele), and effect size, in addition to notes on how the PGS was developed (method used) and applied, as well as assessments of its predictive performance. The GWAS Catalog, a public database with a curated collection of GWAS studies, will also be consulted (53). This catalog will help us to understand the genetic components related to the diseases evaluated, identifying the main SNPs.

Currently, 22 PGS for coronary artery disease (CAD), one for myocardial infarction, and five for ischemic stroke are filed in the bank. We will evaluate the performance of different bank models (with h2SNP >0.05) that were developed and tested in individuals with different ancestry, mainly European, in order to calibrate them to the Brazilian population, whose miscegenation represents a challenge for the generalization of PGS in our population.

In parallel, the need to perform an ancestry estimation through an independent set of SNPs will be evaluated. For each individual, the % contribution of the ancestral component of the 1000G will be estimated (AFR: African, AMR: Native American, EAS: East Asia, EUR: European, and SAS: South Asian) using the ADMIXTURE software (54). This was done to evaluate the relationship between ancestry and potential bias in PGS developed in populations with European ancestry, due to population differences in linkage disequilibrium and allelic frequency, due to genetic drift.

SELECTION OF SNPs FOR PGS CALCULATION

After imputation of the WGS-LP (1x), samples with a genotyping rate >0.99 will be considered and the SNP variants that meet the following 3 criteria will be kept for the PGS calculation: 1) good quality of imputation (INFO>0.8); 2) minor allele frequency (MAF) >1%, and 3) p-value greater than 1x106 in ancestry-specific Hardy-Weinberg equilibrium tests. Ambiguous, mismatched, duplicated SNPs located on sex chromosomes will be discarded (55).

• Rua Treze de Maio, 1.815 • Paulista
• CEP 01327 001 • São Paulo • SP
• Fone: 11 3549 1976 • Fax: 11 3549 0394

For this data quality control assessment, the PLINK tool will be used (56). This tool, implemented in C/C++, allows the manipulation and analysis of GWAS data in an easy and optimized way, acting in several stages, including: data quality control, data management, summary statistics, population stratification, association analysis, and identity-by-descent (IBD) estimation.

PGS CALCULATION AND CALIBRATION

For PGS calculation, the most commonly used method is Clumping and Threshold (C+T), also known as Pruning and Threshold (P+T) (57). Clumping selects the most significant variables iteratively, calculates correlations between nearby variants within a genetic region (w_c), and removes all variants within this region with a correlation value above a threshold defined by the variable r_c^2. Threshold, in turn, consists of removing variants that contain p-values (obtained from the hypothesis test of linear regressions between the number of effect alleles in each sample and the characteristic of interest (51,58)) that exceed the chosen confidence level ($p > p_T$). For each sample, the PGS is then calculated as the sum of the effects found for each risk allele in each SNP. This method is implemented in its standard form by the PRSice-2 tool (55).

O The methods used to calculate the PGS seek to find a statistical compromise between signal and noise. In this context, as the values of the parameters (w_c, r_c^2, p_T) of the C+T method are arbitrary, the method has difficulties in finding the optimal values of the parameters to maximize the quality of the predictions. Thus, other methods were proposed to solve the difficulties presented, such as: methods using LASSO regression (58), Bayesian statistics (59), and machine learning, such as SCT (57,60). Some of these methods can be seen in more detail below:

Lassosum: method used to supplement the PGS calculation with external linkage disequilibrium information (58). It can be used in R or directly from the command line for UNIX systems. In this methodology, based on penalized regression (LASSO), we seek to estimate the effect sizes of the SNPs (the regression coefficients) by minimizing the loss function with a penalty (in this case, $2\lambda\sum_i |\beta_i|$, or the L1 norm of regression β coefficients), usually estimated using cross-validation.

LDPred2: method that estimates mean posterior causal effect sizes from GWAS summary statistics (59). Subsequently, the method filters the variants based on their correlations and similarities associated with other variants in the reference population (61). Thus, an a priori probability is assumed for the genetic architecture and linkage disequilibrium information, enabling the analysis of traits of interest and diseases with a wide range of gene structures. The package methodology is implemented in the bigsnpr package for R (60).

Stacking C+T (SCT): the method uses a penalized regression to find a linear combination of the multiple values of the C+T parameters (57). Like the Bayesian statistical method, SCT is also implemented in the bigsnpr package for R.

MultiPRS: a method developed for the analysis of PGS in mixed populations (62).

The advent of computational biology has brought numerous challenges related to the analysis of large amounts of biological data, storage, and processing. In the scope of GWAS, several tools to facilitate the analyses of SNPs related to characteristics of interest have already been implemented. PLINK, one of the most used tools, calculates the PGS using equation (1) with an adjustment factor in the denominator to weight the potentially missing SNPs (50). This calculation is done as shown in equation (2), where S_i is the effect size of SNP i; G_{ij} is the number of "effect" alleles observed in sample j; P is the ploidy of the sample (2 for humans); N is the total number of SNPs included in the PGS; and M_j is the number of non-missing SNPs in sample j. For samples with missing genotypes for SNP i, population MAF multiplied by ploidy (MAF_i*P) is used instead of G_{ij}.

$$PGS_j=iNS_i*GijP*Mj \qquad (2)$$

In this study, initially, a study of GWAS (Genome-wide association studies) will be carried out for the Brazilian population to obtain the effects of SNPs and external variables (sex, possibly ancestry, age) for each of the clinical conditions. Then, we will use the C+T, LASSO Regression, Bayesian Statistics, and SCT methods to find the best cut-off parameters in the selection of SNPs and perform the PGS calculation for the Brazilian population. The metric used to compare the methods will be based on the area under the curve (AUC) of the resulting ROC curve, sensitivity, and

specificity.

Finally, to minimize the effect of the reduced amount of the sample of the Brazilian population in the calculation of the PGS, we will use a multiethnic PGS through the linear combination between PGS Brazilian population and a PGS European population to be defined, as described in the formula below (62):

$$PGS = \alpha_1 〖PRS〗_{BR} + \alpha_2 〖PRS〗_{EU}$$

where α_1 and α_2 are the weights of the PGS of the Brazilian and European populations, respectively.

The other biochemical tests will be carried out according to a standardized technique by the laboratories of the network associated with the Fleury laboratory (**Table 2**).

• Rua Treze de Maio, 1.815 • Paulista
• CEP 01327 001 • São Paulo • SP
• Fone: 11 3549 1976 • Fax: 11 3549 0394

Table 1: Biochemical tests, stability, and methodology

Test description	ACRONYM	Refrigerated Stability	Frozen Stability	Methodology
Glycated hemoglobin, whole blood	HGBGLIC	(2-8 °C): 7 days;	(-20 °C): 30 days;	Ion exchange Column chromatography in HPLC system
Apolipoprotein A-1, serum	APOLIPA	(2-8 °C): 8 days;	(-20 °C): 2 months;	Immunoturbidimetric
Apolipoprotein B, serum	APOLIPOB	(2-8 °C): 8 days;	(-20 °C): 2 months;	Immunoturbidimetric
Cholesterol, serum	COLEST	(2-8 °C): 7 days;	(-20 °C): 3 months;	Colorimetric enzyme
HDL Cholesterol, serum	HDLCOL	(2-8 °C): 7 days;	(-20 °C): 3 months;	Colorimetric enzyme
Cholesterol, LDL Fraction, serum	LDLTE	(2-8 °C): 7 days;	(-20 °C): 3 months;	Calculation based on Friedewald and Martin formulas
Cholesterol, VLDL Fraction, serum	VLDLTE	(2-8 °C): 7 days;	(-20 °C): 3 months;	Martin and colleagues' formula
Triglycerides, serum	TRIG	(2-8 °C): 7 days;	(-20 °C): 1 year;	Colorimetric enzyme
Creatinine, urine	CREATUR	(2-8 °C): 5 days;	(-20 °C): 1 year.	Colorimetric kinetic
Sodium, serum	NA	(2-8 °C): 7 days;	(-20 °C): 6 months;	Potentiometric
Potassium, serum	K	(2-8 °C): 7 days;	(-20 °C): 6 months;	Potentiometric
SODIUM, SPOT, URINE	NAUR	(2-8 °C): 7 days;	(-20 °C): 6 months;	Potentiometric
Potassium, urine	KUR	(2-8 °C): 7 days;	(-20 °C): 3 months;	Potentiometric
Creatinine, spot, urine	CREATISO	(2-8 °C): 5 days;	(-20 °C): 1 year;	Colorimetric kinetic
Albuminuria, spot, urine	MCRALBISO	(2-8 °C): 14 days;	(-20 °C): 6 months;	Immunoturbidimetric assay

Bias control

Eligibility criteria for cases and controls will be strictly followed to reduce selection bias. The criteria defined for the first CVD event will be standardized and all sites will be trained in the correct identification, data recording, and reporting of information. Despite not being a paired study (to avoid the difficulty of obtaining a high number of controls within the timeframe we have for the study), the relationship between the variables will be adjusted by multivariate logistic models.

The genetic and clinical tests will be carried out by the same network of laboratories, ensuring the standardization of methods.

Sample size

Faced with a complex or polygenic disease model to be studied, i.e., the presence of heterozygous variants added together to represent pathogenic potential, sample size statistical calculations assume a dominant inheritance pattern, with a 1:1 unpaired case-control study design, a sample of 1,867 cases and 1,867 controls would have statistical power of 90% to detect a genetic OR of 1.3, assuming 10% allelic frequency in this 1:1 unpaired control case of genetic factor. A prevalence of 10% of the disease (AMI/CVA/PAD) in the target population was considered, an alpha significance level of 5%, bilateral hypothesis test. Sample size calculation was performed via QUANTO version 1.2.4 (4,5).

Quantitative variables and statistical analysis

The Hardy-Weinberg genetic equilibrium test will be evaluated in the control group using the chi-square test or Fisher's exact test (63). To estimate the association between genetic polymorphisms and risk of cardiovascular disease, univariate and multiple unconditional logistic regression analyses will be conducted (64–66).

Initially, univariate binary logistic regression analyses will be conducted. Then, covariates with p-value < 0.20 in univariate regression analyses will be considered in multiple logistic regression analysis with selection of variables according to the backward elimination technique (65,66). P-value < 0.05 will be used as a retention criterion for variables in the model.

In backward selection, a variant of backward elimination that considers the maximum possible sample size as the number of covariates in the model decreases at each step of this iterative process will be used (67). Occasionally, covariates judged as confounding factors by the investigator may be forced into the final backward regression model.

Additional multiple logistic regression analyses may also be conducted using a variant of the Purposeful Selection algorithm described by Bursac *et al* (68,69).

The assumption of linearity on the logit scale (log-odds) between each quantitative covariate and the binary response variable in binary logistic regression analysis will be evaluated with the construction of "Smoothed Scatter Plots" and the method of fractional polynomials (65,70). When the assumption is not satisfied, quantitative covariates will be categorized for use in logistic regression using cut-off points, according to the literature, distribution tertiles or optimal cut-off obtained from the Receiver Operating Characteristic (ROC) Curve, whichever is deemed most appropriate. In the case of an optimal cut-off point, it will be defined as the one that maximizes the Youden index (70,71).

The presence of multicollinearity will be evaluated through the estimation of variance inflation factors ("Variance Inflation Factor" (VIF)). VIF values > 2.5 will serve as indicators of considerable multicollinearity in logistic regression analysis (65). Gross and adjusted odds ratios (OR) with 95% CI will be reported.

Possible interactions between covariates that remain in the final regression model will be evaluated, including interaction

• Rua Treze de Maio, 1.815 • Paulista
• CEP 01327 001 • São Paulo • SP
• Fone: 11 3549 1976 • Fax: 11 3549 0394

terms in the model (64,65,72).

The calibration and discriminatory ability of the final multiple logistic regression model will be evaluated via the Hosmer-Lemeshow test and the area under the ROC curve (AROC), respectively. P-value > 0.05 for the Hosmer-Lemeshow test indicates that the model would be calibrated, that is, the probabilities predicted by the model adequately reflect the occurrence of the event in the data. As a general rule, the following interpretation will be considered for the AROC: AROC=0.5: absent discrimination, 0.5<=AROC<0.7: minor discrimination, 0.7<=AROC<0.8: acceptable discrimination, 0.8<=AROC<0.9: excellent discrimination; AROC>=0.9: nearly perfect discrimination (66).

Risk Attributable to Population will be estimated.

In the characterization of the study population, categorical variables will be described with counts and proportions and compared with Pearson's chi-square test or with Fisher's exact test (73). Normally and asymmetrically distributed quantitative variables will be described as mean (standard deviation) or median (interquartile range), respectively (74). Normality will be assessed by visually inspecting histograms and applying normality tests, if appropriate (75,76). Comparison of these continuous type variables will be carried out with Student's t test for independent samples or non-parametric alternative, Mann-Whitney test (73).

All statistical analyses will follow the complete case analysis principle. All hypothesis tests will be bilateral and p-value<0.05 considered statistically significant. Statistical data analysis will be conducted with SAS 9.4 (SAS Institute, Cary, NC).

Ethical issues

This study will follow all the recommendations of good practice and ethics in research, as established in CNS Resolution No. 466 of 2012, in Document of the Americas, and other relevant legislation. All participants must sign an informed consent form (ICF), respecting all rights of the study participants, including secrecy and confidentiality. The research will be explained to all patients and, at any time, the right to withdraw consent will be assured.

Considering that polygenic CVDs may have little explanation related to the genetic issue, disclosing the data in advance, without proper adjustments for phenotypic, clinical, and demographic variables, may be hasty, and may generate fuss, dropouts, and even a lack of credibility in this research. Therefore, the risk data attributable to genetics will be released at the end of the research when they are properly adjusted (see data source and measurement section above).

The project will be submitted to the HAOC's EC as ethical coordinator.

• Rua Treze de Maio, 1.815 • Paulista
• CEP 01327 001 • São Paulo • SP
• Fone: 11 3549 1976 • Fax: 11 3549 0394
