# Supplementary material for: Impact of genetic background as a risk factor for atherosclerotic cardiovascular disease: A protocol for a nationwide genetic case-control (CV-GENES) study in Brazil
Source: PLoS One. 2024 Mar 13;19(3):e0289439. doi: 10.1371/journal.pone.0289439 (PMC10936812; doi:10.1371/journal.pone.0289439)
Supplement: S3 File — (DOCX) [file pone.0289439.s003.docx]

**Supplementary material 3**

**Eligibility Criteria**

**1. Acute myocardial infarction**

A case of acute MI will be validated if met the current criteria of an acute myocardial injury with clinical evidence of acute myocardial ischemia, and with detection of a rise and/or fall of cardiac troponin (TnI or TnT) values with at least 1 value above the 99th percentile URL and at least 1 of the following: [^1^](#_ENREF_1)

• Symptoms of myocardial ischemia;

• New ischemic ECG changes;

• Development of pathological Q waves;

• Imaging evidence of new loss of viable myocardium or new regional wall motion abnormality in a pattern consistent with an ischemic etiology;

• Identification of a coronary thrombus by angiography

**2. Stroke**

A case of an acute stroke will be validated by the following: CNS infarction is brain, spinal cord, or retinal cell death attributable to ischemia, based on a) clinical evidence of cerebral, spinal cord, or retinal focal ischemic injury based on symptoms persisting ≥24 hours or until death, and other etiologies are excluded. (Note: CNS infarction includes hemorrhagic infarctions, types I and II); b) pathological, imaging, or other objective evidence of cerebral, spinal cord, or retinal focal ischemic injury in a defined vascular distribution. Therefore, an acute onset of signs and/or symptoms suspected to be due to vascular origin, prior medical history, clinical examination plus imaging evidence of diagnostic tests such as CT scan, MRI scan, angioCT, angioMRI etc, are the foundation to confirm the diagnosis of an acute stroke.[^2^](#_ENREF_2)

**3. Peripheral artery thrombotic or ischemic events**

A case of an acute peripheral artery ischemic/thrombotic event will be validated when a patient present with symptoms that develop in several minutes, to hours or days (up to 14 days for clinical diagnosis of an acute versus subacute or chronic event) and range from new or worsening intermittent claudication to severe rest pain, paresthesia, muscle weakness, paralysis and even gangrene. The classical description of patients with acute limb ischemia is grouped into a mnemonic known as the “6 Ps”: pain, pallor, paralysis, pulse deficit, paresthesia and poikilothermia. Diagnostic imaging includes Duplex Ultrasound, Computed Tomography Angiography (CTA) and Magnetic Resonance Angiography (MRA), or Invasive Angiogram. [^3^](#_ENREF_3)^,^ [^4^](#_ENREF_4)

**Genetic Component Test**

*DNA EXTRACTION*

DNA from blood samples will be extracted by an automated process (QIASymphony, using the QIAmp DNA Mini Kit – Qiagen) following the manufacturers’ instructions. The extracted DNA will be quantified by the Qubit Fluorometer (Thermo Fisher) and will be kept at -20 °C until its use.

*PREPARATION OF THE EXOME AND LOW PASS 1x GENOME LIBRARIES AND SEQUENCING*

For the preparation of the whole exome sequencing and low pass whole genome sequencing, the paired-ends libraries will be prepared using 50 ng of DNA as input and enzymatic fragmentation and combinatorial dual indexes (Twist Bioscience) reagent kit, following the manufacturer´s instructions.

Exome and low pass genome will be sequenced on a NovaSeq 6000 platform (IlIumina) using S4 flow cell with 300 cycles (2 x 150 bp - paired end). Run data and quality control will be monitored in NovaSeq control software.

*ANALYSIS OF THE GENERATED DATA*

The generated data will be processed in two distinct pipelines. The Exome pipeline is already deployed and functional. The pipeline that will process the low-pass WGS is also functional, and the imputation module is under validation. Both pipelines were validated accordingly to GATK Best Practices recommendation [^5^](#_ENREF_5)^,^ [^6^](#_ENREF_6) and Dragen DNA Pipeline (Illumina®).[^7^](#_ENREF_7)

Both pipelines are based on Illumina’s Dragen v.3.10 system installed on servers located at Fleury Group’s headquarters. At the end of the runs, the pipeline will perform the demultiplexing step, creating the FASTQ files (containing the reads) for each sample, aligning the reads to the reference human genome GRCh37 (hg19), creating the BAM files containing the aligned reads and creating the VCFs files containing the identified variants (SNPs, INDELS, CNV). After completing this step, the generated BAM and VCF files will be stored in the cloud (AWS) and then sent to the Emedgene platform (Illumina) for annotation/classification of variants found in the genes: *ABCG5*, *ABCG8*, *APOB*, *APOE, LDLR*, *LDLRAP1*, *LIPA, PCSK9*. All data generated will be processed, analyzed, and reported by specialized professionals (bioinformaticians, analysts, and geneticists). Low-pass 1x genome data will be submitted to the imputation method, which guarantees quality in the identification of variants and will be used with the call of exome variants by the systems and data team for PRS calibration.

*CALIBRATION AND CALCULATION OF PRS FOR HEART DISEASES*

The literature on PRS calculation is relatively recent. Despite this, its evolution grows at an accelerated pace, mainly due to the increase in genetic datasets that allow the proposition of new calculation methods and the growth of its use for disease prediction, as a result of the increase in its accuracy in research based on cases control and population in general. [^8^](#_ENREF_8)

PRS aggregates the effects of genetic variants into a single number that predicts genetic predisposition to a phenotype. PRS are typically composed of hundreds to millions of genetic variants (usually SNPs). For each individual, the number of risk alleles computed in each variant is summed and weighed by the estimated value of the effects obtained (log odds ratio for traits with binary values or Beta coefficients for traits with continuous value (68), the risk alleles are selected from a Genome Wide Association Study (GWAS) performed with the case and controls patients, as can be seen in equation 1:

PRS= β_i k_i+ β_(i+1) k_(i+1)+⋯ +β_n k_n (1)

where β_i,β_(i+1),…,β_n are the effects obtained, k_i,k_(i+1),…,k_n the number of risk alleles computed in each variant (0, 1 or 2), and i,i+1,…,n are the indices of the SNPs (51).

*SELECTION AND EVALUATION OF PRS MODELS*

For the calculation and calibration of PRS for heart diseases, previous PRS models will be selected from the PGS Catalog. [^9^](#_ENREF_9) The PGS Catalog is an open database of published polygenic scores (PRS). Each PRS is consistently annotated with relevant metadata including score files, which contain information on variants, risk alleles (effect allele), and effect size, in addition to notes on how the PRS was developed (method used) and applied, as well as assessments of its predictive performance. The GWAS Catalog, a public database with a curated collection of GWAS studies, will also be consulted.[^10^](#_ENREF_10) This catalog will help us to understand the genetic components related to the diseases evaluated, identifying the main SNPs.

Currently, 22 PRS for coronary artery disease (CAD), one for myocardial infarction, and five for ischemic stroke are filed in the bank. We will evaluate the performance of different bank models (with h2SNP >0.05) that were developed and tested in individuals with different ancestry, mainly European, in order to calibrate them to the Brazilian population, whose miscegenation represents a challenge for the generalization of PRS in our population.

In parallel, the need to perform an ancestry estimation through an independent set of SNPs will be evaluated. For each individual, the % contribution of the ancestral component of the 1000G will be estimated (AFR: African, AMR: Native American, EAS: East Asia, EUR: European, and SAS: South Asian) using the ADMIXTURE software. This will be done to evaluate the relationship between ancestry and potential bias in PRS developed in populations with European ancestry, due to population differences in linkage disequilibrium and allelic frequency, due to genetic drift.

*SELECTION OF SNPs FOR PRS CALCULATION*

After imputation of the WGS-LP (1x), samples with a genotyping rate >0.99 will be considered and the SNP variants that meet the following 3 criteria will be kept for the PRS calculation: 1) good quality of imputation (INFO>0.8); 2) minor allele frequency (MAF) >1%, and 3) p-value greater than 1x106 in ancestry-specific Hardy-Weinberg equilibrium tests. Ambiguous, mismatched, duplicated SNPs located on sex chromosomes will be discarded.[^11^](#_ENREF_11)

For this data quality control assessment, the PLINK tool will be used. [^12^](#_ENREF_12)This tool, implemented in C/C++, allows the manipulation and analysis of GWAS data in an easy and optimized way, acting in several stages, including data quality control, data management, summary statistics, population stratification, association analysis, and identity-by-descent (IBD) estimation.

*PRS CALCULATION AND CALIBRATION*

For PRS calculation, the most commonly used method is Clumping and Threshold (C+T), also known as Pruning and Threshold (P+T). [^13^](#_ENREF_13) Clumping selects the most significant variables iteratively, calculates correlations between nearby variants within a genetic region (w_c), and removes all variants within this region with a correlation value above a threshold defined by the variable r_c^2. Threshold, in turn, consists of removing variants that contain p-values (obtained from the hypothesis test of linear regressions between the number of effect alleles in each sample and the characteristic of interest [^14^](#_ENREF_14)^,^ [^15^](#_ENREF_15) that exceed the chosen confidence level (p > p_T). For each sample, the PRS is then calculated as the sum of the effects found for each risk allele in each SNP. This method is implemented in its standard form by the PRSice-2 tool.[^12^](#_ENREF_12)

The methods used to calculate the PRS seek to find a statistical compromise between signal and noise. In this context, as the values of the parameters (w_c, r_c^2, p_T) of the C+T method are arbitrary, the method has difficulties in finding the optimal values of the parameters to maximize the quality of the predictions. Thus, other methods were proposed to solve the difficulties presented, such as: methods using LASSO regression[^15^](#_ENREF_15), Bayesian statistics[^16^](#_ENREF_16), and machine learning, such as SCT[^13^](#_ENREF_13)^,^ [^17^](#_ENREF_17). Some of these methods can be seen in more detail below:

Lassosum: method used to supplement the PRS calculation with external linkage disequilibrium information. [^15^](#_ENREF_15) It can be used in R or directly from the command line for UNIX systems. In this methodology, based on penalized regression (LASSO), we seek to estimate the effect sizes of the SNPs (the regression coefficients) by minimizing the loss function with a penalty (in this case, 2λ∑_i▒〖|β_i |〗, or the L1 norm of regression β coefficients), usually estimated using cross-validation.

LDPred2: method that estimates mean posterior causal effect sizes from GWAS summary statistics.[^16^](#_ENREF_16) Subsequently, the method filters the variants based on their correlations and similarities associated with other variants in the reference population[^18^](#_ENREF_18). Thus, an a priori probability is assumed for the genetic architecture and linkage disequilibrium information, enabling the analysis of traits of interest and diseases with a wide range of gene structures. The package methodology is implemented in the bigsnpr package for R [^13^](#_ENREF_13).

Stacking C+T (SCT): the method uses a penalized regression to find a linear combination of the multiple values of the C+T parameters [^19^](#_ENREF_19). Like the Bayesian statistical method, SCT is also implemented in the bigsnpr package for R.

MultiPRS: a method developed for the analysis of PRS in mixed populations.[^20^](#_ENREF_20)

The advent of computational biology has brought numerous challenges related to the analysis of large amounts of biological data, storage, and processing. In the scope of GWAS, several tools to facilitate the analyses of SNPs related to characteristics of interest have already been implemented. PLINK, one of the most used tools, calculates the PRS using equation (1) with an adjustment factor in the denominator to weight the potentially missing SNPs [^8^](#_ENREF_8).This calculation is done as shown in equation (2), where S_i is the effect size of SNP i; G_ij is the number of “effect” alleles observed in sample j; P is the ploidy of the sample (2 for humans); N is the total number of SNPs included in the PRS; and M_j is the number of non-missing SNPs in sample j. For samples with missing genotypes for SNP i, population MAF multiplied by ploidy (〖MAF〗_i*P) is used instead of G_ij.

〖PRS〗_j=iNS_i*GijP*Mj (2)

In this study, initially, a study of GWAS (Genome-wide association studies) will be carried out for the Brazilian population to obtain the effects of SNPs and external variables (sex, possibly ancestry, age) for each of the clinical conditions. Then, we will use the C+T, LASSO Regression, Bayesian Statistics, and SCT methods to find the best cut-off parameters in the selection of SNPs and perform the PRS calculation for the Brazilian population. The metric used to compare the methods will be based on the area under the curve (AUC) of the resulting ROC curve, sensitivity, and specificity.

Finally, to minimize the effect of the reduced amount of the sample of the Brazilian population in the calculation of the PRS, we will use a multiethnic PRS through the linear combination between PRS Brazilian population and a PRS European population to be defined, as described in the formula below[^20^](#_ENREF_20):

PRS= α_1 〖PRS〗_BR+ α_2 〖PRS〗_EU

where α_1 and α_2 are the weights of the PRS of the Brazilian and European populations, respectively.

The other biochemical tests will be carried out according to a standardized technique by the laboratories of the network associated with the Central laboratory (**table1**)

**Supplementary Material Reference**

1. Thygesen K, Alpert JS, Jaffe AS, Chaitman BR, Bax JJ, Morrow DA, et al. Fourth universal definition of myocardial infarction (2018). European heart journal 2019;40(3):237-269.

2. Sacco RL, Kasner SE, Broderick JP, Caplan LR, Connors J, Culebras A, et al. An updated definition of stroke for the 21st century: a statement for healthcare professionals from the American Heart Association/American Stroke Association. Stroke 2013;44(7):2064-2089.

3. Callum K, Bradbury A. Acute limb ischaemia. Bmj 2000;320(7237):764-767.

4. Olinic D-M, Stanek A, Tătaru D-A, Homorodean C, Olinic M. Acute limb ischemia: an update on diagnosis and management. Journal of clinical medicine 2019;8(8):1215.

5. Van der Auwera GA, Carneiro MO, Hartl C, Poplin R, Del Angel G, Levy‐Moonshine A, et al. From FastQ data to high‐confidence variant calls: the genome analysis toolkit best practices pipeline. Current protocols in bioinformatics 2013;43(1):11.10. 1-11.10. 33.

6. DePristo MA, Banks E, Poplin R, Garimella KV, Maguire JR, Hartl C, et al. A framework for variation discovery and genotyping using next-generation DNA sequencing data. Nature genetics 2011;43(5):491-498.

7. Illumina. DRAGEN DNA Pipeline. In.

8. Lewis CM, Vassos E. Polygenic risk scores: from research tools to clinical instruments. Genome medicine 2020;12(1):1-11.

9. Lambert SA, Gil L, Jupp S, Ritchie SC, Xu Y, Buniello A, et al. The Polygenic Score Catalog as an open database for reproducibility and systematic evaluation. Nature genetics 2021;53(4):420-425.

10. Buniello A, MacArthur JAL, Cerezo M, Harris LW, Hayhurst J, Malangone C, et al. The NHGRI-EBI GWAS Catalog of published genome-wide association studies, targeted arrays and summary statistics 2019. Nucleic acids research 2019;47(D1):D1005-D1012.

11. Choi SW, Mak TS-H, O’Reilly PF. Tutorial: a guide to performing polygenic risk score analyses. Nature protocols 2020;15(9):2759-2772.

12. Purcell S, Neale B, Todd-Brown K, Thomas L, Ferreira MA, Bender D, et al. PLINK: a tool set for whole-genome association and population-based linkage analyses. The American journal of human genetics 2007;81(3):559-575.

13. Privé F, Aschard H, Ziyatdinov A, Blum MG. Efficient analysis of large-scale genome-wide data with two R packages: bigstatsr and bigsnpr. Bioinformatics 2018;34(16):2781-2787.

14. de Villiers CB, Kroese M, Moorthie S. Understanding polygenic models, their development and the potential application of polygenic scores in healthcare. Journal of medical genetics 2020;57(11):725-732.

15. Mak TSH, Porsch RM, Choi SW, Zhou X, Sham PC. Polygenic scores via penalized regression on summary statistics. Genetic epidemiology 2017;41(6):469-480.

16. Privé F, Arbel J, Vilhjálmsson BJ. LDpred2: better, faster, stronger. Bioinformatics 2020;36(22-23):5424-5431.

17. Tachmazidou I, Süveges D, Min JL, Ritchie GR, Steinberg J, Walter K, et al. Whole-genome sequencing coupled to imputation discovers genetic signals for anthropometric traits. The American journal of human genetics 2017;100(6):865-884.

18. Khera AV, Chaffin M, Aragam KG, Haas ME, Roselli C, Choi SH, et al. Genome-wide polygenic scores for common diseases identify individuals with risk equivalent to monogenic mutations. Nature genetics 2018;50(9):1219-1224.

19. Privé F, Vilhjálmsson BJ, Aschard H, Blum MG. Making the most of clumping and thresholding for polygenic scores. The American journal of human genetics 2019;105(6):1213-1221.

20. Márquez‐Luna C, Loh PR, Consortium SATD, Consortium STD, Price AL. Multiethnic polygenic risk scores improve risk prediction in diverse populations. Genetic epidemiology 2017;41(8):811-823.
